# Cyclase-associated protein (CAP) inhibits inverted formin 2 (INF2) to induce dendritic spine maturation

**DOI:** 10.1007/s00018-024-05393-y

**Published:** 2024-08-18

**Authors:** Cara Schuldt, Sharof Khudayberdiev, Ben-David Chandra, Uwe Linne, Marco B. Rust

**Affiliations:** 1 Molecular Neurobiology Group, Institute of Physiological Chemistry, Philipps-University of Marburg, 35032 Marburg, Germany; 2 Center for Mind, Brain and Behavior (CMBB), University of Marburg and Justus-Liebig-University Giessen, 35032 Marburg, Germany; 3 Department of Chemistry, Philipps-University Marburg, 35032 Marburg, Germany

**Keywords:** Spinogenesis, Synaptogenesis, Synapse formation, Actin acetylation, Acetylated actin

## Abstract

The morphology of dendritic spines, the postsynaptic compartment of most excitatory synapses, decisively modulates the function of neuronal circuits as also evident from human brain disorders associated with altered spine density or morphology. Actin filaments (F-actin) form the backbone of spines, and a number of actin-binding proteins (ABP) have been implicated in shaping the cytoskeleton in mature spines. Instead, only little is known about the mechanisms that control the reorganization from unbranched F-actin of immature spines to the complex, highly branched cytoskeleton of mature spines. Here, we demonstrate impaired spine maturation in hippocampal neurons upon genetic inactivation of cyclase-associated protein 1 (CAP1) and CAP2, but not of CAP1 or CAP2 alone. We found a similar spine maturation defect upon overactivation of inverted formin 2 (INF2), a nucleator of unbranched F-actin with hitherto unknown synaptic function. While INF2 overactivation failed in altering spine density or morphology in CAP-deficient neurons, INF2 inactivation largely rescued their spine defects. From our data we conclude that CAPs inhibit INF2 to induce spine maturation. Since we previously showed that CAPs promote cofilin1-mediated cytoskeletal remodeling in mature spines, we identified them as a molecular switch that control transition from filopodia-like to mature spines.

**Supplementary Information:**

The online version contains supplementary material available at 10.1007/s00018-024-05393-y.

## Introduction

Most excitatory synapses of the vertebrate brain are formed on small dendritic protrusions termed dendritic spines [7, 62]. Dendritic spines own a postsynaptic density (PSD) that opposes the presynaptic active zone and contains neurotransmitter receptors, ion channels and signaling molecules that collectively mediate postsynaptic response to neurotransmitter release [62]. Scaffolding proteins anchor the PSD to actin filaments (F-actin), the major cytoskeletal component that define spine morphology and thereby modulate synapse physiology and brain function. Spines are initially formed as filopodia-like protrusions mainly consisting of unbranched F-actin. Upon axonal contact, they transform into thin spines that own a tiny head-like structure composed of branched F-actin [28, 39, 42]. During further maturation, the branched actin cytoskeleton expands to form stubby and mushroom-like spines, which both have large bulbous heads, but differ in neck length and width [30, 39]. Mature spines are highly plastic and change their morphology in response to neuronal activity, thereby contributing decisively to long-term potentiation (LTP) and depression (LTD), two major forms of synaptic plasticity that underlie brain functions like learning and memory [7, 29, 38, 44, 71].

Spine formation, maturation and plasticity depend on precise cytoskeletal organization, which requires the coordinated activity of actin-binding proteins (ABP) [28, 30, 39, 42]. To date, only few ABP have been implicated in the formation of filopodia-like spines including mouse diaphanous 2 (mDia2), a formin family member that nucleates unbranched F-actin, and the elongation factor vasodilator-stimulated phosphoprotein [31, 39, 41]. Instead, numerous ABP have been identified as morphological regulators of mature spines [7, 70], of which actin-related protein 2/3 (Arp2/3) complex, the major nucleator of branched F-actin [50], and the F-actin disassembly factor ADF/cofilin emerged as main actors [6, 23, 51, 53, 64]. Conversely, very little is known about the mechanisms that control cytoskeletal reorganization during transition from filopodia-like into mature spines [30, 39].

In vitro studies of the past decade identified cyclase-associated protein (CAP) as actin regulators that cooperates with ADF/cofilin to accelerate subunit dissociation from F-actin pointed ends [33, 36, 37, 61]. In line with these studies, we demonstrated that CAP1 accelerates actin dynamics in growth cones or dendritic spines together with cofilin1 [26, 57, 59], the major ADF/cofilin family member in the brain [6, 21, 23, 31, 51, 53, 56, 68, 74]. More recently, we identified CAP1 as a regulator of neuronal gene expression that represses activity of the transcription factor serum response factor (SRF) and its co-activator myocardin-related transcription factor (MRTF) by an actin-dependent mechanism [35]. Different to most other species, vertebrates own a second family member, CAP2, with restricted tissue distribution and established functions in striated muscles [13, 18, 34, 69]. The vertebrate brain is unique in that it is the only tissue expressing two CAP family members at substantial levels [54, 55]. However, their brain functions are only poorly understood, and it remained unknown whether CAP1 and CAP2 acquired different or redundant functions.

By exploiting mouse hippocampal neurons, we demonstrate that genetic inactivation of both CAPs severely impaired spine maturation. We found a similar spine maturation defect upon overactivation of inverted formin 2 (INF2), a formin family member with hitherto unknown synaptic functions. INF2 physically interacts with both CAPs in hippocampal cells, and INF2 overactivation did not alter spine morphology in CAP-deficient neurons, while its genetic depletion rescued their spine defects. These findings and our previous studies let us propose a model in which CAP1 and CAP2 control spine maturation by inhibiting INF2 and by promoting cofilin1, an established actin regulator in mature spines [26, 46, 51].

## Results

### CAP2 inactivation does not alter dendritic spine density or size

**Fig. 1:**
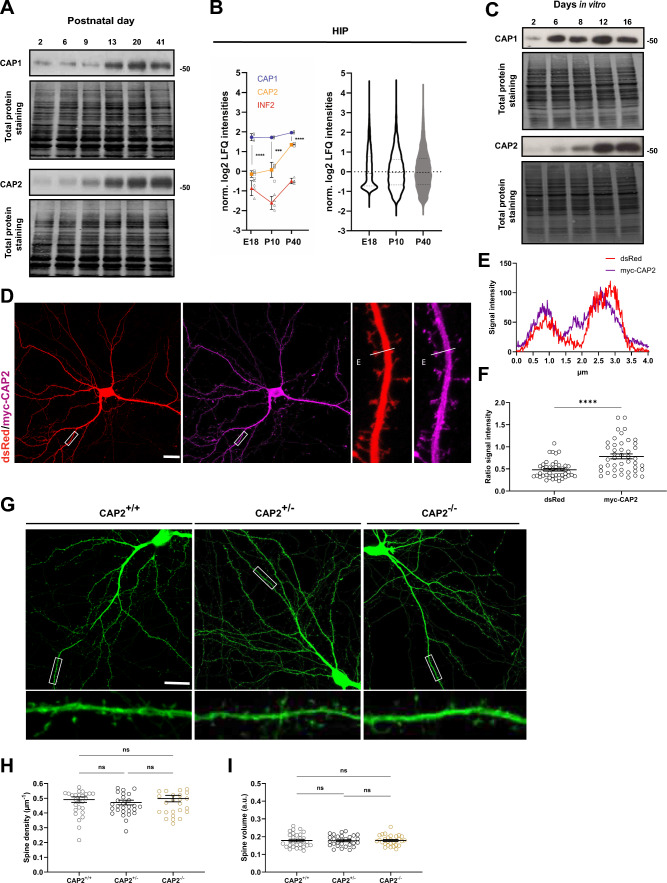
CAP2 inactivation does not alter dendritic spine density or size. **A** Immunoblots showing CAP1 and CAP2 expression throughout postnatal hippocampus development. Total protein staining was performed to confirm equal loading. **B** Expression levels of CAP1, CAP2 and INF2 determined by mass spectrometry on hippocampal lysates from E18.5, P10 and P40 mice. Graph includes values of individual protein samples (N = 4), mean values (MV) and standard error of the means (SEM). Statistical comparison of CAP1 and CAP2 expression levels were performed using Student’s t-test and corrected for multiple comparison with Bonferroni method. Violin plots to the right show expression level distribution of all proteins in these lysates. Norm. log2 LFQ intensity of zero indicates median expression level of all proteins detected within respective condition. **C** Immunoblots showing CAP1 and CAP2 expression in isolated cortical neurons. Total protein staining was performed to confirm equal loading. **D** Micrograph of a representative DIV16 hippocampal neuron expressing dsRed (red) together with myc-CAP2 (magenta). Box indicate area shown with higher magnification. **E** Fluorescence intensity profiles for dsRed and myc-CAP2 immunoreactivity along white line in D. **F** Graph showing ratio of spine head/dendritic shaft for dsRed and myc-CAP2. Graph includes values of individual spines, MV and SEM. 45 spines from 15 neurons and 3 biological replicates have been investigated (N = 45/15/3). Student’s t-test was performed to test for statistical significance. **G** Representative micrographs of GFP-expressing DIV16 hippocampal neurons from littermates of various genotype. Graphs showing **H** spine density and **I** spine volume in neurons from wildtype mice (CAP2^+/+^), heterozygous CAP2 mutants (CAP2^+/-^) and systemic CAP2 mutants (CAP2^−/−^). Scale bars: 20 µm. ns: P > 0.05, ***: P < 0.001, ****: P < 0.0001

To compare expression of CAP1 and CAP2 during hippocampus development, we performed immunoblots on same protein lysates. This analysis confirmed increasing CAP1 expression throughout postnatal development [26], and it revealed a similar increase for CAP2 (Fig. 1A). To compare their absolute expression levels, we performed mass spectrometry on hippocampal and cerebral cortex lysates at embryonic day 18.5 (E18.5), postnatal day 10 (P10) and P40 (Figs. 1B, S1A-B, Tables S1, S2). This analysis confirmed developmental increases in the expression of both CAPs. Compared to CAP2, CAP1 levels were roughly fourfold higher in both tissues at E18.5 (log2 converted values for HIP: CAP1 1.73 ± 0.18, CAP2 –0.15 ± 0.17, P < 0.0001; CTX: CAP1 1.65 ± 0.03, CAP2 –0.09 ± 0.08, P < 0.0001), and they remained higher at P40 (log2 converted values for HIP: CAP1 1.96 ± 0.06, CAP2 1.34 ± 0.06, P < 0.0001; CTX: CAP1 2.01 ± 0.07, CAP2 1.52 ± 0.07, P < 0.001). Abundance of both ABP in adult forebrain was confirmed by in situ hybridization (Fig. S1C-D). We also determined their expression in isolated neurons, which revealed that both increased during differentiation (Fig. 1C). In line with its established function in early differentiation processes [57, 58], CAP1 levels strongly increased soon after plating. Instead, CAP2 expression substantially increased at later stages, during the 2nd week in culture. Together, both CAPs are expressed in the hippocampus and expression of both increased during neuron differentiation. However, CAP1 levels were higher when compared to CAP2, whose expression increased at later differentiation stages.

We previously demonstrated postsynaptic localization of endogenous CAP1 and CAP2 in differentiated neurons as well as an enrichment of myc- or green fluorescent protein (GFP)-tagged CAP1 in dendritic spines [25, 26, 46]. To test whether CAP2 was similarly enriched in dendritic spines, we transfected hippocampal neurons at day 6 in vitro (DIV6) with myc-tagged CAP2 (myc-CAP2) and the volume marker *Discosoma* red fluorescent protein (dsRed). Myc antibody staining in DIV16 neurons revealed presence of myc-CAP2 in spines, as also evident from its fluorescence intensity profile (Fig. 1D-E). Moreover, the ratio of myc immunoreactivity in spine heads versus dendritic shafts was increased when compared to the ratio of dsRed (Fig. 1F). These data confirmed postsynaptic CAP2 localization and revealed enrichment of myc-CAP2 in spines.

Postsynaptic CAP2 localization let us investigate whether it controls spine density or morphology, similar to CAP1 [25, 26]. To do so, we exploited hippocampal neurons that we isolated from E18.5 systemic CAP2 mutant mice (CAP2^−/−^), whose generation has been described before [34]. Immunoblots confirmed efficient CAP2 inactivation and unaltered CAP1 expression in hippocampus from CAP2^−/−^ mice (Fig. S1E-G). We compared the morphology of neurons from CAP2^−/−^ mice to neurons from heterozygous (CAP2^+/-^) or wildtype (CAP2^+/+^) littermates. At DIV6, neurons were transfected with the volume marker GFP that allowed us to examine morphology at DIV16 (Fig. 1G). Neither spine density nor spine GFP intensity, a parameter frequently used to assess spine size [25, 26], was different between neurons isolated from CAP2^−/−^, CAP2^+/-^ or CAP2^+/+^ mice (Fig.  1H-I). Hence, hippocampal neurons isolated from CAP2^−/−^ mice did not display changes in spine density or size, different from CAP1-deficient neurons.

### CAP1 and CAP2 share overlapping functions in dendritic spines

Normal spine density and size in CAP2^−/−^ neurons and higher CAP1 expressions levels prompted us to test whether CAP1 can compensate for CAP2 loss. We therefore generated neurons lacking both CAPs and compared their morphology to neurons lacking either CAP1 or CAP2. To do so, we isolated hippocampal neurons from CAP1^flx/flx^/CAP2^−/−^ and CAP1^flx/flx^/CAP2^+/-^ embryos and transfected them at DIV6 with GFP together with either catalytically active mCherry-tagged Cre recombinase (Cre) or a catalytically inactive mCherry-Cre (Cre-mut). We thereby obtained (i) Cre-expressing CAP1^flx/flx^/CAP2^−/−^ neurons (termed double knockout (dKO)), (ii) Cre-mut-expressing CAP1^flx/flx^/CAP2^−/−^ neurons (termed CAP2-KO), (iii) Cre-expressing CAP1^flx/flx^/CAP2^+/-^ neurons (for simplicity termed CAP1-KO) and Cre-mut-expressing CAP1^flx/flx^/CAP2^+/-^ neurons that served as controls (CTR). Previous studies demonstrated efficient CAP1 inactivation in CAP1^flx/flx^ neurons upon Cre expression and normal CAP1 levels upon Cre-mut expression [26, 35, 57], thereby validating suitability of our approach.

**Fig. 2:**
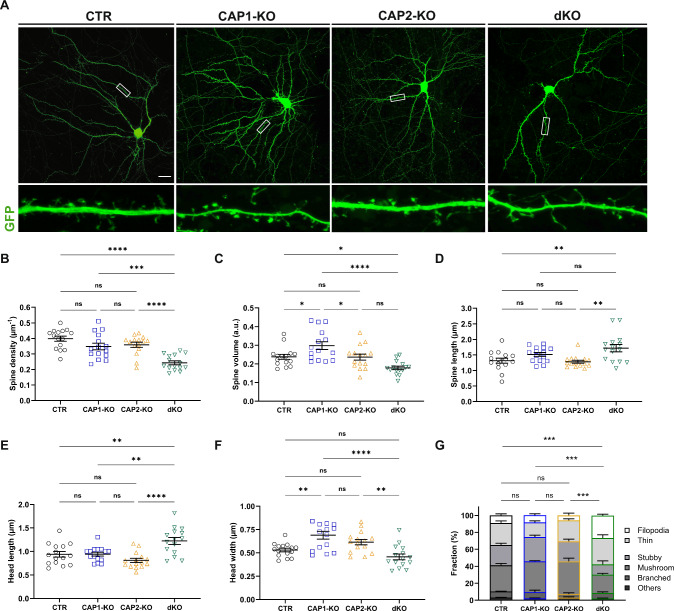
Spine morphological changes in DIV16 dKO neurons lacking CAP1 and CAP2. **A** Micrographs of representative DIV16 neurons expressing GFP. Boxes indicate areas shown at higher magnification. Graphs showing **B** spine density, **C** spine volume, **D** spine length, **E** head length, **F** head width and **G** fraction of spine types. Scale bar (µm): 20. ns: P ≥ 0.05, *: P < 0.05, **: P < 0.01, ***: P < 0.001, ****: P < 0.0001

In line with our analyses in neurons isolated from CAP2^−/−^ mice (Fig. 1G-I) and our previous study in CAP1-deficient neurons [26], spine density and size were unchanged in CAP2-KO neurons, while CAP1-KO neurons displayed an increased spine size (Fig. 2A-C). Instead, dKO neurons displayed strong reductions in spine density and size, which were decreased by 40% and 20%, respectively, when compared to CTR neurons. We noted similarly reduced spine density (–32%) and size (–20%) in an independent experiment, in which we acutely inactivated both CAPs by transfecting DIV6 CAP1^flx/flx^ neurons with Cre and a shRNA against CAP2 (Fig. S2A-C), whose efficiency has been validated by nucleofection in cortical neurons (Fig. S2D). In these experiments, CAP1^flx/flx^ neurons transfected with Cre-mut and control shRNA (CTR-sh) served as controls.

A more detailed spine morphometric analysis revealed roughly 30% increases in spine length and spine head length in dKO neurons, while spine head width was not different from CTR neurons (Fig. 2D-F). None of these parameters were changed in CAP2-KO neurons, and CAP1-KO solely displayed a 30% increase in spine head width. Similar to previous studies [26, 27], we categorized spines according to their morphologies (Fig. S3A). Compared to CTR neurons, the morphologies of filopodia-like, thin, stubby and mushroom-like spines was largely unchanged in neurons lacking either CAP1, CAP2 or both (Fig. S3B-E). Further, the fractions of spine types were not different between CTR and single KO neurons (Fig. 2G). Conversely, the fraction of filopodia-like spines was 2.3-fold higher in dKO neurons, and the thin spine fraction increased by roughly 25%. Instead, the fractions of stubby and mushroom-like spines were reduced by roughly 50% and 35%, respectively. Consequently, the spine type distribution in dKO neurons was shifted towards smaller spines, which usually represent the postsynaptic compartments of immature synapses.

**Fig. 3:**
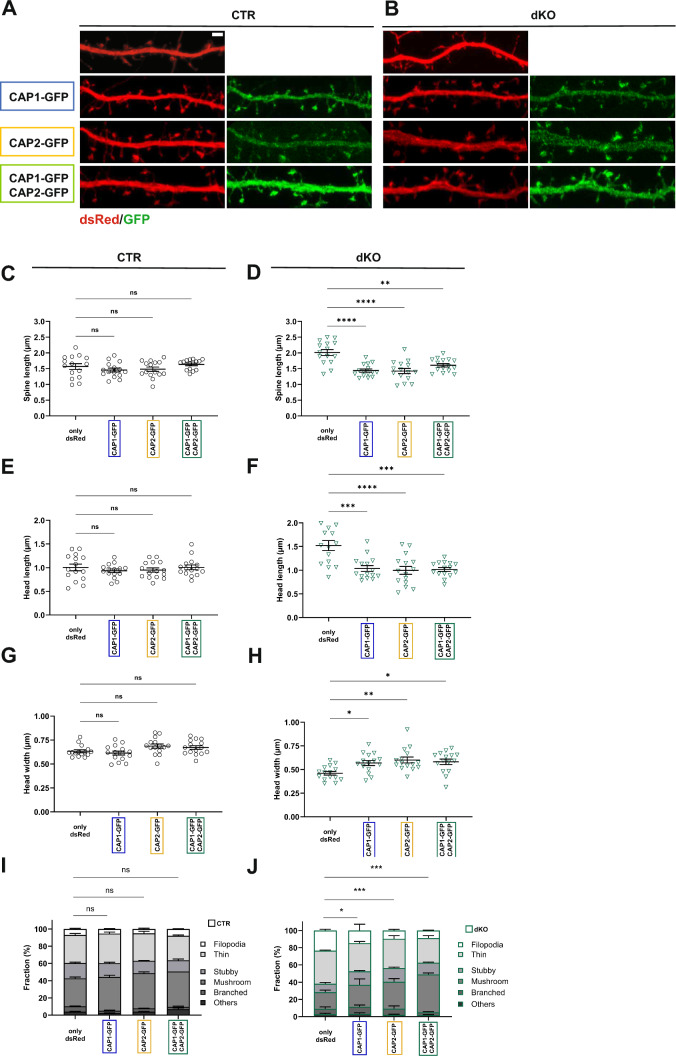
Expression of CAP1 or CAP2 is sufficient to rescue spine morphological changes in DIV16 dKO neurons. Micrographs of representative dendritic shafts from **A** CTR neurons or **B** dKO neurons expressing either dsRed only or dsRed together with either CAP1-GFP or CAP2-GFP or CAP1-GFP and CAP2-GFP. Graphs showing **C-D** spine length, **E–F** head length, **G-H** head width and **I-J** fraction of spine types in CTR and dKO neurons upon expression of either CAP1-GFP, CAP2-GFP or both. Scale bar (µm): 2. ns: P ≥ 0.05, *: P < 0.05, **: P < 0.01, ***: P < 0.001, ****: P < 0.0001

Together, dKO neurons displayed spine morphological changes characterized by strongly reduced spine density, increased spine length and a shift towards an immature spine profile. These changes were not present in single KO neurons, thereby suggesting overlapping functions for CAP1 and CAP2 in spines. Indeed, overexpression of either CAP1-GFP or CAP2-GFP was sufficient to normalize spine morphology and spine type distribution in dKO neurons (Fig. 3A-J). Instead, neither overexpression of CAP1-GFP nor of CAP2-GFP or both changed spine morphology or spine type distribution in CTR neurons. Together, our data demonstrated that CAP1 and CAP2 acquired overlapping functions in regulating spine morphology.

### Impaired spine maturation upon compound inactivation of CAP1 and CAP2

**Fig. 4:**
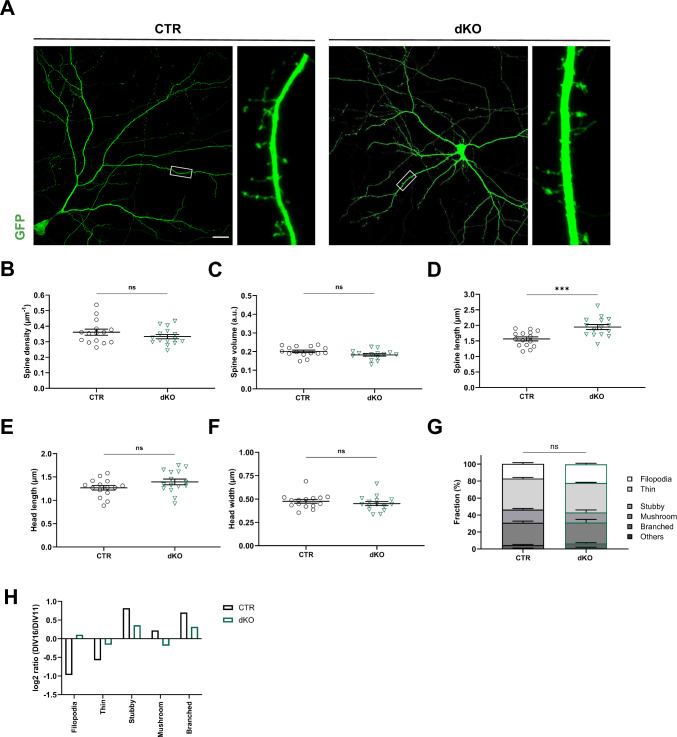
Overall normal spine morphology in DIV11 dKO neurons. **A** Micrographs of representative DIV11 CTR and dKO neurons expressing GFP. Boxes indicate areas shown at higher magnification. Graphs showing **B** spine density, **C** spine volume, **D** spine length, **E** head length, **F** head width and **G** fraction of spine types in both groups. **H** Log2 values for DIV16/DIV11 ratio of spine types in CTR and dKO neurons. Scale bar (µm): 20. ns: P ≥ 0.05, ***: P < 0.001

Next, we examined dKO neurons at an earlier stage to narrow down onset of spine defects. To do so, we compared the morphology of GFP-transfected CTR and dKO neurons at DIV11 (Fig. 4A). Different from DIV16, spine density and spine size were both unchanged in DIV11 dKO neurons (Fig. 4B-C). While spine length was increased by 25%, all other parameters including spine head length, spine head width as well as the morphologies of spine types did not differ between CTR and dKO neurons at DIV11 (Figs. 4D-F, S4), just as the fractions of spine types (Fig. 4G). Together, spine morphology was overall similar between DIV11 CTR and dKO neurons, thereby identifying DIV11 as a stage before onset of spine changes in dKO neurons.

Further, we compared spine type fractions between DIV11 and DIV16 for both groups. In line with the expected spine maturation in CTR neurons, this comparison revealed developmental decreases in the fractions of small spines (filopodia-like, thin), while the fractions of stubby, mushroom-like and branched spines increased (Fig. 4H). Consequently, spine type distribution shifted towards a more mature profile between DIV11 and DIV16 CTR neurons (comparison of CTR neurons shown in Figs. 4G and 2G, P < 0.0001). Instead, we did not note such developmental changes in dKO neurons, and the spine type distribution was not different between DIV11 and DIV16 (comparison of dKO neurons shown in Figs. 4G and 2G, P = 0.73). These data suggested a failure in spine maturation between DIV11 and DIV16 in dKO neurons. We therefore concluded that CAP1 and CAP2 acquired overlapping postsynaptic functions that became relevant during spine maturation.

### INF2 is located in spines and interacts with CAP1 and CAP2

Having demonstrated overlapping functions for CAP1 and CAP2 in spine maturation, we next set out to unravel the underlying mechanism. For the following reasons, we decided to study the function of INF2 in dendritic spines: (i) it belongs to the formin family that nucleates unbranched F-actin, (ii) formins have been implicated in outgrowth of filopodia-like spines, whose cytoskeleton is mainly composed of unbranched F-actin, (iii) a complex of actin and CAP purified from mouse brain was shown to inhibit INF2, and (iv) CAP1 and CAP2 acquired similar INF2 inhibitory functions [1, 2, 30, 31, 39]. We therefore hypothesized that loss of both CAPs caused an overactivation of INF2 that contributes to spine defects in dKO neurons.

**Fig. 5:**
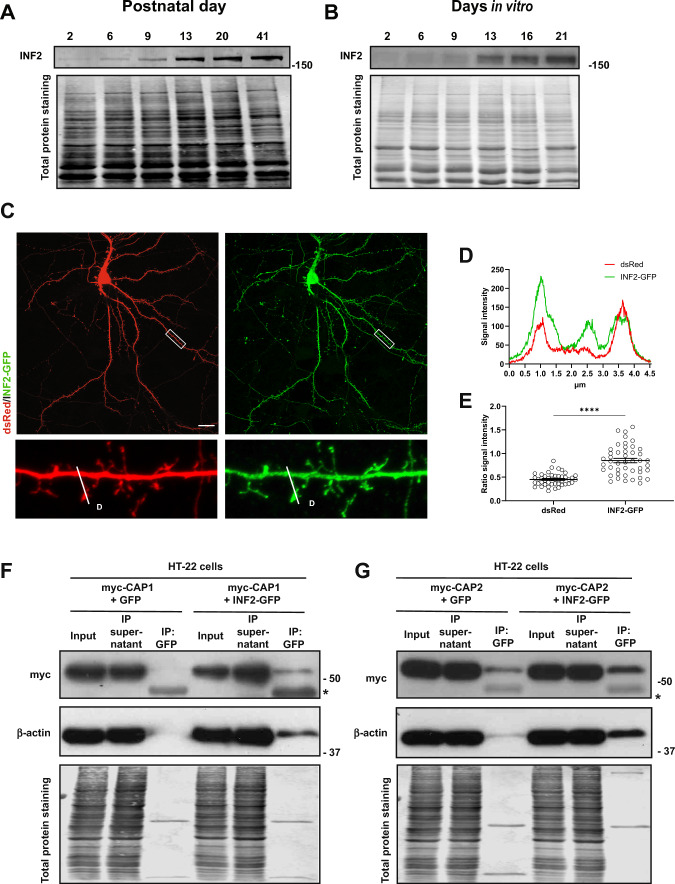
INF2 is located in spines and interacts with CAP1 and CAP2 in neuronal cells. Immunoblots showing INF2 expression **A** in postnatal hippocampus and **B** cultured cortical neurons. Total protein staining was performed to control equal loading. **C** DIV16 hippocampal neuron expressing dsRed (red) together with INF2-GFP (green). Box indicate area shown with higher magnification. **D** Fluorescence intensity profiles for dsRed and INF2-GFP along white line in C. **E** Graph showing ratio of spine head/dendritic shaft for dsRed and INF2-GFP. Graph includes values of individual spines and MV ± SEM, N = 45/15/3. Student’s t-test was performed to test for statistical significance. **F** Immunoblots with antibody against myc and β-actin in lysates from HT-22 cells expressing either myc-CAP1 and GFP or myc-CAP1 and INF2-GFP. *: lower bands correspond to the heavy chain of IgG. **G** Immunoblots with antibody against myc in lysates from HT-22 cells expressing either myc-CAP2 and GFP or myc-CAP2 and INF2-GFP. Scale bar (µm): 20. ****: P < 0.0001

To date, only little is known about the function of INF2 in the brain [11], and its expression, neuronal localization or synaptic function has not yet been investigated. Immunoblots revealed increasing INF2 expression throughout postnatal hippocampus development (Fig. 5A). Mass spectrometry confirmed a developmental increase of INF2 expression in the postnatal brain (Figs. 1B, S1B), and in situ hybridization expression in adult hippocampus (Fig. S5A). Moreover, INF2 expression increased during differentiation of isolated neurons, particularly displaying a huge increase during 2nd week in culture (Fig. 5B). Further, we found GFP-tagged INF2 (INF2-GFP) to be enriched in spines (Fig. 5C), which was evident from both fluorescence intensity profile of INF2-GFP in DIV16 neurons and its ratio in spine head versus dendritic shaft (Fig. 5D-E).

Moreover, by co-immunoprecipitation (CoIP) we showed interaction of INF2 with both CAPs in the hippocampus-derived cell line HT-22. In these experiments, we overexpressed myc-CAP1 or myc-CAP2 together with either GFP or INF2-GFP, and we precipitated myc-CAP1 and myc-CAP2 with a GFP antibody in lysates from INF2-GFP-expressing cells (Figs. 5F-G, S5B-C). Instead, no myc-CAP1 and only a very small amount of myc-CAP2 was precipitated with the GFP antibody in lysates from HT-22 cells expressing myc-CAP1 or myc-CAP2 together with GFP, which served as negative controls. Notably, only small amounts of myc-CAP1 or myc-CAP2 were present in the IP fractions, while most myc-CAP1 and myc-CAP2 did not bind INF2-GFP. Similarly, only a small fraction of endogenous β-actin was present in the IP fractions (Fig. 5F-G). Together, our data revealed that (i) INF2 expression increased during neuron differentiation at about the period spine defects became apparent in dKO neurons, (ii) INF2 was enriched in spines and (iii) INF2 interacted with both CAP1 and CAP2 in neuronal cells.

### Overactivation of INF2 phenocopied spine defects of dKO neurons

**Fig. 6:**
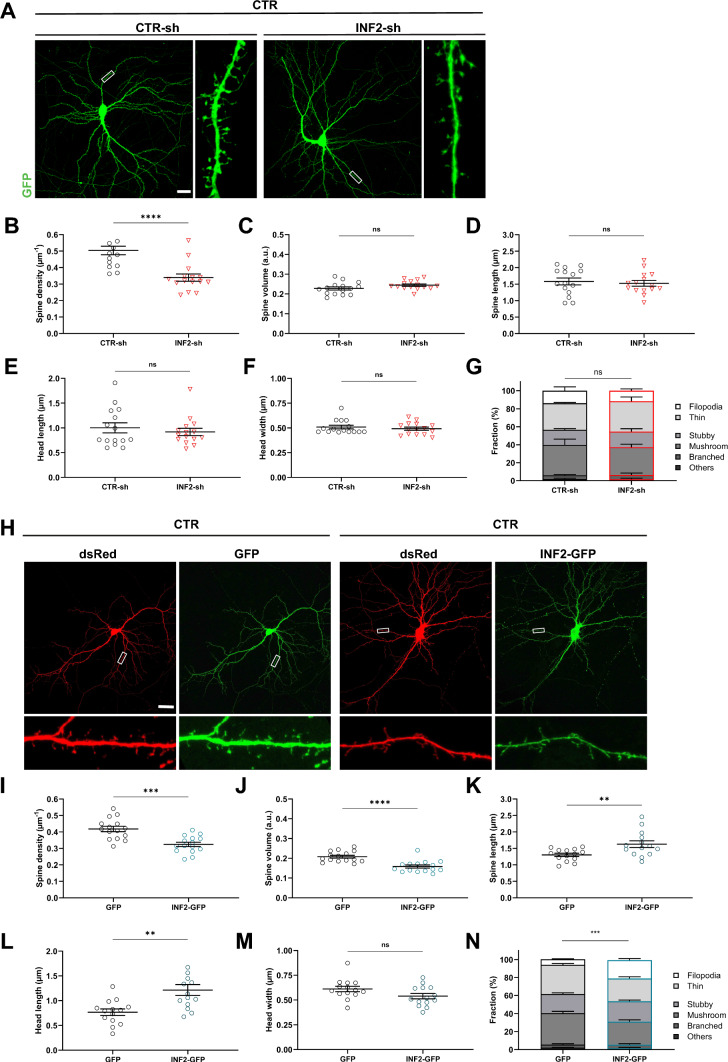
INF2 overactivation phenocopied spine defects of dKO neurons. **A** Micrographs of CTR neurons expressing GFP (green) together with either CTR-sh or INF2-sh. Boxes indicate areas shown at higher magnification. Graphs showing** B** spine density, **C** spine volume, **D** spine length, **E** head length, **F** head width and **G** fraction of spine types in CTR neurons expressing either CTR-sh or INF2-sh. **H** Micrographs of representative CTR neurons expressing dsRed (red) together with either GFP or INF2-GFP (green). Boxes indicate areas shown at higher magnification. Graphs showing **I** spine density, **J** spine volume, **K** spine length, **L** head length, **M** head width and **N** fraction of spine types in CTR neurons expressing either GFP or INF2-GFP. Scale bars (µm): 20. ns: P ≥ 0.05, **: P < 0.01, ***: P < 0.001, ****: P < 0.0001

Increasing expression during neuron differentiation and postsynaptic localization of INF2 led us hypothesize that INF2 is functionally relevant in dendritic spines. To test this hypothesis, we induced both inactivation and overactivation of INF2 in CTR neurons. To inactivate INF2, we designed a shRNA directed against INF2 (INF2-shRNA), for which immunoblots validated efficient knockdown (INF2-KD) in HT-22 cells (Fig. S6A-B). We transfected DIV6 CTR neurons with GFP together with either INF2-shRNA or CTR-shRNA, and determined spine density and morphology at DIV16 (Fig. 6A). When compared to CTR-shRNA-transfected (INF2-CTR) neurons, spine density was reduced by 35% in INF2-KD neurons (Fig. 6B), while spine volume, spine length, spine head length and spine head width was unchanged (Fig. 6C-F). We also compared the morphologies and fractions of spine types between INF2-CTR and INF2-KD neurons and did not find differences between both groups (Figs. 6G, S6C-F). Together, INF2 inactivation in hippocampal neurons reduced the density, but not overall morphology of dendritic spines, nonetheless demonstrating functional relevance for INF2 in dendritic spines.

To increase INF2 activity, we transfected CTR neurons with INF2-GFP together with the volume marker dsRed at DIV6 and determined spine density and morphology at DIV16 (Fig. 6H). Compared to neurons expressing dsRed and GFP, which served as controls in these experiments, spine density was reduced by 22% and spine volume by 24% upon INF2-GFP expression (Fig. 6I-J). Further, expression of INF2-GFP increased spine length by + 25% and spine head length by + 38% (Fig. 6K-L). Instead, spine head width was slightly lower upon INF2-GFP expression (Fig. 6M), but this decrease did not reach statistical significance. While the morphologies of spine types were not different between CTR neurons expressing either GFP or INF2-GFP (Fig. S6G-J), INF2-GFP expression increased the filopodia-like spine fraction and reduced the mushroom-like spine fraction (Fig. 6N), thereby shifting the spine type distribution towards a more immature spine profile. Taken together, these data implicated INF2 in the regulation of spine density and morphology. Further, they revealed that INF2-GFP overactivation induced spine morphological changes in CTR neurons resembling those of dKO neurons lacking CAP1 and CAP2. Our data thereby support our hypotheses that INF2 was dysregulated in dKO neurons and that INF2 overactivation contributes to their spine defects.

### INF2 inhibition rescues spine maturation defect in CAP-deficient neurons

**Fig. 7:**
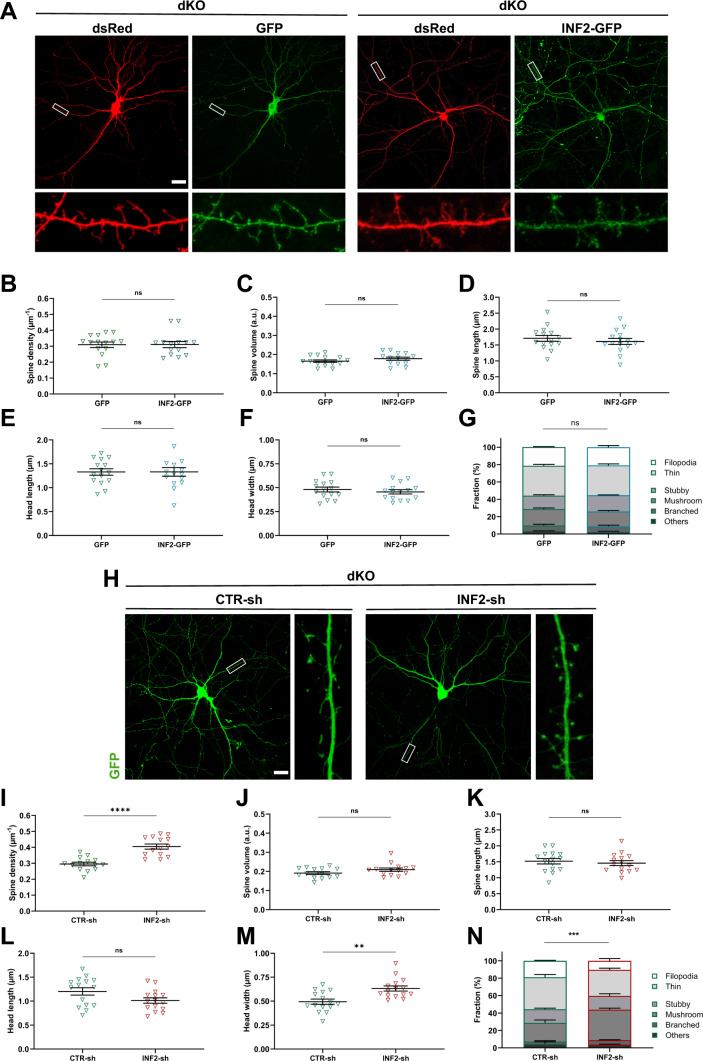
INF2 inhibition rescues spine maturation defect in dKO neurons. **A** Micrographs of representative dKO neurons expressing dsRed (red) together with either GFP or INF2-GFP (green). Boxes indicate areas shown at higher magnification. Graphs showing** B** spine density, **C** spine volume, **D** spine length, **E** head length, **F** head width and **G** fraction of spine types in dKO neurons expressing either GFP or INF2-GFP. **H** Micrographs of representative dKO neurons expressing GFP (green) together with either CTR-sh or INF2-sh. Boxes indicate areas shown at higher magnification. Graphs showing **I** spine density, **J** spine volume, **K** spine length, **L** head length, **M** head width and **N** fraction of spine types in dKO neurons upon transfection of either CTR-sh or INF2-sh. Scale bars (µm): 20. ns: P ≥ 0.05, **: P < 0.01, ***: P < 0.001, ****: P < 0.0001

To test these hypotheses, we first transfected dKO neurons with dsRed together with either GFP or INF2-GFP (Fig. 7A). Compared to GFP-expressing dKO neurons, spine density and volume were both unchanged in dKO neurons upon expression of INF2-GFP (Fig. 7B-C). Similarly, INF2-GFP expression neither changed spine length, spine head length and spine head width nor the morphologies or fractions of spine types (Figs. 7D-G S7A-D). Together, INF2 overactivation failed in altering spine density or morphology in dKO neurons. This was in stark contrast to CTR neurons, in which INF2 overactivation reduced spine density and induced an immature spine profile.

Finally, we tested whether INF2 inhibition rescues spine defects in dKO neurons. We therefore transfected DIV6 dKO neurons with GFP together with either INF2-sh or CTR-sh, and determined spine density and morphology at DIV16 (Figs. 7H–N, S7E-H). When compared to CTR-sh-transfected dKO neurons, spine volume and length was unchanged in dKO neurons upon INF2 inactivation. However, spine density was increased by roughly 30% (Fig.  7I). Upon INF2 inactivation, spine density in dKO neurons was not different from spine density in CTR neurons (shown in Fig. 2B, P =  0.07). Importantly, INF2 inactivation reduced the fractions of filopodia-like and thin spines in dKO neurons, while it increased the fraction of mushroom-like spines, thereby shifting the spine type distribution in dKO neurons towards a mature profile. Upon expression of INF2-sh, spine type distribution in dKO neurons was not different from that in CTR neurons (shown in Fig. 2G, P = 0.346). Together, shRNA-mediated INF2 inactivation rescued spine changes in neurons lacking CAP1 and CAP2, thereby demonstrating that INF2 overactivation caused spine defects in dKO neurons, particularly the reduced spine density as well as the shift towards an immature spine profile.

## Discussion

By exploiting primary neurons from gene targeted mice, we demonstrated overlapping postsynaptic functions for CAP1 and CAP2, two ABP with largely unknown physiological functions. Compound inactivation of both, but not of CAP1 or CAP2 alone, caused strongly reduced spine density and an immature spine profile in hippocampal neurons. We noted very similar changes upon overactivation of INF2, an ABP with hitherto unknown synaptic function, for which we reported postsynaptic localization and interaction with CAP1 and CAP2. Further, we found spine defects in neurons lacking CAP1 and CAP2 largely rescued upon INF2 inhibition. These findings let us propose a novel mechanism relevant for the maturation of dendritic spines, in which CAPs antagonize INF2 activity to allow transition from filopodia-like to mature spines.

Different to most invertebrate species, vertebrates own two CAP family members that are highly homologous and possess similar protein domains, but differ in their tissue distribution [54, 55]. While CAP1 is broadly expressed including brain, CAP2 expression is abundant in heart, skeletal muscle and brain. In line with its enrichment in striated muscles, CAP2^−/−^ mice displayed skeletal muscle and cardiac defects [13, 18, 34, 45, 69], and human genetic studies associated pathogenic *CAP2* variants with nemaline myopathy or dilated cardiomyopathy [5, 12, 24, 55]. Instead, CAP2 functions in the brain largely remained elusive, also because brain defects have not been reported for CAP2^−/−^ mice or associated with human *CAP2* mutations. We here showed unchanged spine density and morphology in hippocampal neurons isolated from CAP2^−/−^ mice, while a previous study reported normal spine density and slight increases in spine length and width upon shRNA-mediated CAP2 knockdown in rat hippocampal neurons [46]. This discrepancy could be explained by compensatory mechanisms in CAP2^−/−^ neurons, which did not come into effect upon acute inactivation.

While CAP2 inactivation did not compromise neuron morphology, we previously demonstrated that genetic CAP1 inactivation impaired growth cone function and gene expression in differentiating neurons, thereby compromising neuronal connectivity in CAP1-KO brains [35, 57], and that CAP1 inactivation in differentiated neurons enlarged dendritic spines and impaired structural plasticity [25, 26]. Hence, CAP1 emerged as the major family member in the vertebrate brain, and it therefore remained elusive why this tissue is unique in that it expresses both CAPs at substantial levels.

Absence of obvious defects in CAP2^−/−^ neurons or brains, but severe defects upon CAP1 inactivation could be explained by their expression levels, since we found fourfold higher CAP1 expression in hippocampus and cerebral cortex at late embryonic stages, which was still higher in adult mice. We therefore assumed that CAP1 can compensate for CAP2 loss, whereas CAP2 cannot compensate CAP1 loss. CAP2 expression strongly increased in brain lysates and isolated neurons during a time period critical for synaptogenesis [72, 75], and this rise coincided with the onset of spine defects in dKO neurons including reduced spine density and a shift towards an immature spine profile. These defects were not present in single KO neurons, thereby demonstrating overlapping postsynaptic functions for CAP1 and CAP2. Confirmatively, expression of either CAP1-GFP or CAP2-GFP was sufficient to rescue spine defects in dKO neurons. Overlapping functions of both CAP family members is in line with our previous studies, in which we rescued defects in growth cone morphology, neuron differentiation and neuronal gene expression in CAP1-KO neurons by expression of myc- or GFP-tagged CAP2 [35, 58]. Hence, different effects of CAP1 and CAP2 inactivation in neurons or brain can be explained less by functional differences than by their expression levels.

In the present study, we not only demonstrated overlapping functions for both CAPs in dendritic spines, but also unraveled a postsynaptic function for INF2, an ABP with largely unknown functions in the brain, for which a protective role in ischemia-induced neuronal death has only recently been reported [11]. We found developmentally increased INF2 expression in hippocampal lysates and isolated neurons as well as postsynaptic abundance of INF2-GFP. Further, we showed that INF2 overactivation reduced spine density and that it strongly increased the fraction of filopodia-like spines and reduced the fraction of mushroom-like spines, thereby inducing an immature spine profile in hippocampal neurons. The latter finding suggested that INF2 promotes the formation of filopodia-like spines, in which the cytoskeleton is mainly composed of unbranched F-actin [30, 39]. Such a function is in good agreement with its primary molecular activity, i.e. nucleation of unbranched F-actin [9]. Hence, INF2 has acquired a postsynaptic function similar to mDia2, another formin that has been implicated in the formation and elongation of filopodia-like spines [31]. We have thus provided evidence that formation of filopodia-like spines does not only depend on only a single formin, in line with previous assumptions [30].

While INF2-GFP expression induced an immature spine profile in CTR neurons, it failed in altering spine morphology in dKO neurons. This could be explained by an intrinsic INF2 overactivation in dKO neurons, which was not further increasable by ectopic INF2 expression. In line with this idea, neurons either overexpressing INF2 or lacking both CAPs displayed similar spine morphological changes, and reduced spine density and the shift towards an immature spine profile in dKO neurons was rescued upon shRNA-mediated INF2 inactivation. Further, it is in very good agreement with recent studies that revealed antagonistic functions of CAPs for formin-mediated F-actin polymerization. In vitro experiments showed that mouse CAP1 was able to displace the FH2 domain of mDia1 from of F-actin’s barbed ends [4]. However, this study did not investigate whether it was able to similarly displace INF2, and this finding has been challenged by a recent study that determined the effect of the yeast CAP homolog SRV2 on barbed end residence time of the FH2 domain [66]. More relevant for the present study, an inhibitory function towards INF2 has been demonstrated for a protein complex consisting of actin and CAP1 and/or CAP2 [1, 2]. Data of the latter two studies led to a model in which actin/CAP complexes facilitate INF2 autoinhibition by serving as a bridge between its N-terminal diaphanous inhibitory domain (DID) and C-terminal diaphanous autoregulatory domain (DAD) [3]. In line with these studies, we here confirmed by CoIP interaction between INF2 and both CAPs in neuronal cells. Interestingly, the aforementioned studies reported similar INF2 inhibitory activities for both CAP1 and CAP2, which likely explains absence of INF2 overactivation and, hence, of immature spine profiles in single KO neurons lacking either one or the other family member. While these studies unraveled a novel mechanism of INF2 inhibition, the physiological relevance of the INF2 interaction with actin/CAP complexes remained unknown [1, 2]. However, the authors speculated that compromised interaction of INF2 with actin/CAP complexes may contribute to the mechanisms underlying human diseases such as focal segmental glomerulosclerosis and Charcot-Marie-Tooth [2], which both have been linked to dominant missense mutations in INF2’s DID that reportedly binds CAPs with sub-micromolar affinity [1, 2, 8, 10]. We here report a physiological function for the INF2-CAP interaction by demonstrating that it is relevant for spine maturation. Further, our data led us speculate that dysregulated INF2-CAP interaction may contribute to human neuropsychiatric disorders that have been associated with both impaired spine maturation and dysregulation of the postsynaptic actin cytoskeleton [17, 19, 20, 47, 49, 64].

The inhibitory activity of actin/CAP complexes towards INF2 depended on lysine acetylation in actin (KAc-actin) as acetylation of K50 and K61 strongly increased the affinity of actin/CAP complexes for INF2 [1, 2]. These studies have thus not only unraveled a novel INF2 regulatory mechanism, but also emphasized the relevance of posttranslational modifications (PTMs) of actin. Although PTMs of actin is a rapidly expanding area in cytoskeleton research [14, 67], still only little is known about their physiological relevance and in particular about the physiological relevance of actin acetylation in the nervous system. Interestingly, the INF2 inhibitory KAc-actin/CAP complex has been initially purified from mouse brain, and disease-linked INF2 mutations (including mutations linked to Charcot-Marie-Tooth neuropathy) were only poorly inhibited by this complex [2], suggesting a disease contribution of this interaction [3]. Further, a recent human genetic study associated mutations in *NAA80*, which encodes an enzyme specific for N-acetylation of actin [15], among others with actin dysregulation, brain developmental defects and intellectual disabilities [43]. Collectively, these studies suggest important functions for actin acetylation in the nervous system. Our data are in line with a model, in which KAc-actin/CAP complexes inhibit INF2 in excitatory synapses to induce spine maturation, thereby further supporting the relevance of actin acetylation in the brain. In our CoIP experiments, we only found a small fraction of myc-CAP1, myc-CAP2 or endogenous actin to be co-precipitated with INF2-GFP, which may indicate that in neurons only a minor fraction of actin is present as KAc-actin.

While our data are in line with an INF2 inhibitory function of CAP1 and CAP2 during spine maturation, we previously implicated CAP1 in actin regulation in mature spines [26]. Further, we showed that CAP1 controls spine morphology in cooperation with cofilin1, a key postsynaptic actin regulator relevant for synaptic plasticity, learning and memory [6, 22, 23, 31, 51–53, 65, 68, 74], whose dysregulation has been implicated in various neuropsychiatric disorders [16, 48, 63, 73]. Spine morphological changes upon inactivation of either CAP1 or cofilin1 are probably due to an expansion of the branched F-actin network in spine heads. Moreover, we showed functional interdependence of CAP1 and cofilin1 in the control of spine morphology [26] and that CAP2-dependent postsynaptic cofilin1 recruitment was required for spine remodeling during LTP [46]. Taken together, these studies and our present data revealed that CAPs exert two different postsynaptic functions relevant for distinct developmental steps in spines: CAPs inhibit INF2, a formin family member that can nucleate and prolong unbranched F-actin, which we here implicated in the formation of filopodia-like spines. Further, they promote cofilin1-dependent reorganization of the branched actin cytoskeleton in mature spines. We therefore propose that CAPs act as a molecular switch that controls the balance between unbranched and branched F-actin in spines, which is relevant for the transition from filopodia-like spines into mature spines.

## Material and methods

### Transgenic mice

Mice were housed in the animal facility of the University of Marburg on 12-h dark–light cycles with food and water available ad libitum. Generation of CAP1^flx/flx^ mice have been described before [57], CAP2^−/−^ mice have been obtained from European Conditional Mouse Mutagenesis Program (EUCOMM).

### In situ hybridization

In situ hybridization data on male P56 C57Bl/6 J mice were extracted from the Allen Mouse Brain Atlas (https://mouse.brain-map.org/), originally described by Lein and colleagues [40]. URLs of images shown in Figs. S1C-D and S5 are provided in Table S3*.*

### Cell culture and transfection

Primary hippocampal neurons from embryonic day 18.5 (E18.5) mice were isolated as previously described [60]. Briefly, after dissociation of hippocampi neurons were plated on 0.1 mg/ml poly-L-lysine-coated coverslips at a density of 62,000/cm^2^ and cultured in Neurobasal medium containing 2% B27, 1 mM GlutaMax, 100 µg/ml streptomycin, and 100 U/ml penicillin (Gibco, Thermo Fischer) in a 24 well plate. Neurons were stored in a humidified incubator at 37 °C with 5% CO_2_. DIV6 neurons were transfected with a total amount of 1 μg plasmid/well of 24-well plates using Lipofectamine 2000 reagent (Thermo Fisher) according to manufacturer’s protocol. In all experiments, the same amount of each individual construct has been transfected. Empty pcDNA3.1 vector has been added to set total DNA amount to the desired quantity.

HT-22 cells were plated at a density of 10,000 cells/cm^2^ in 6 well plates. Cells were transfected 24 h after plating with 20 µg plasmid/dish using Lipofectamine 2000 reagent (Thermo Fischer). After 3 days, HT-22 cells were lysed using 1,5 mL ice-cooled lysis buffer (50 mM Tris/HCl pH 7.5, 150 mM NaCl, 1% Triton-X, Roche Proteinase inhibitors) and shaken for 15 min at 4 °C. Afterwards, cells were harvested by scraping and used for further analysis.

### Spine analysis

Images were acquired with Leica TCS SP5 II LSM and LAS AF software using a 63 × oil immersion objective with a resolution of 2,048 × 2,048 pixels as z-stacks of 8 optical planes (step size of 0.49 μm) and projected to a single-plane image (maximum projection). In order to quantify spine density and volume, 150–220 individual spines from secondary and tertiary basal dendrites per neuron were tagged with a circle (FIJI’oval ‘ tool, 2.254 µm^2^ size). To determine spine density, the length of the analyzed dendritic section was measured using the ‘freehand line’ tool. Mean signal intensity in the circle was used to analyze spine volume. Mean signal intensity of all analyzed spines was calculated for every neuron and normalized to mean signal intensity of the respective dendritic shafts. Five neurons of three independent biological replicates were analyzed. Spine morphology (total length, spine head length and width) was analyzed of images of dendritic sections of about 30 µm length from secondary and tertiary basal dendrites using the FIJI ‘freehand selection’ tool. Spines were categorized as shown in scheme in Fig. S3A according to the measured morphometric values. Spines that did not fit into these categories were classified as ‘others’. Five neurons of three independent biological replicates were analyzed. Intensity profiles in confocal images were acquired with FIJI ‘plot profile’ tool. Lines were selected in a way that they cover one mushroom-like spines and the underneath dendritic shaft. GFP ratios between spine head versus dendritic shaft were analyzed with ‘freehand selection’ tool. Like this, mean signal intensities in spine head or an underlying piece of dendrite was determined, which were used to calculate the ratios of mean signal intensities.

### Immunocytochemistry

At DIV11 or DIV16, neurons were washed once with Neurobasal medium. Afterwards, neurons were fixed in 4% paraformaldehyde (PFA)/sucrose in phosphate-buffered saline (PBS) for 15 min and washed three times for five minutes in PBS. After 10 min incubation in carrier solution (0.1% gelatin, 0.3% Triton-X100 in PBS), neurons were incubated with primary antibodies in carrier solution for 2 h. After washing with PBS three times for 5 min, neurons were incubated with secondary antibodies in carrier solution for 60 min. After three five-minute washing steps in PBS, coverslips were mounted onto microscopy slides using Aqua-Poly/Mount (Polysciences Inc.). Primary and secondary antibodies including their dilutions are listed in tables S4 and S5.

### Protein extraction and immunoblots

Prefrontal cortices were snap frozen in liquid N_2_ and stored at –80 °C. Using a dounce homogenizer, 6–10 strokes were performed for homogenization in 750 µl lysis buffer (50 mM Tris-pH 7.5, 150 mM NaCl, 1% Triton-X100, 1 × Complete Protease Inhibitor Cocktail, Roche) and followed by 20 min centrifugation. 100 µl supernatant was collected and used for further analysis. Protein lysates from isolated neurons were generated from five coverslips (250,000 neurons/coverslip). Neurons were treated with with 50 µl lysis buffer incl. PST (1 × PhosSTOP, Roche) on ice. After 10 min incubation on the shaker at 4 °C, neurons were lysed by pipetting 10 times up and down. Afterwards, proteins were separated on a 10% SDS-PAGE PAGE and transferred o/n at 4 °C and 27 V onto a poly-vinylidene difluoride membrane (GE Healthcare) using Mini-Protean electrophoresis system (Biorad). Tris-buffered saline containing 5% milk powder and 0.2% Tween 20 (TBS-T/milk) was used to prevent non-specific antibody binding. Membranes were incubated with primary antibody diluted in TBST-T/milk dilutions for 2 h at RT on the shaker. After washing for three with TBS-T/milk, membranes were incubated with horseradish peroxidase-conjugated secondary antibodies (Thermo Fisher Scientific) in TBS-T/milk for 1 h on the shaker. Before developing with Amersham ECLplus reagent (GE Healthcare), Membranes were washed three times with TBS-T for 10 min. Primary and secondary antibodies including their dilutions are listed in tables S4 and S5.

### Co-immunoprecipitation

HT-22 cells were seeded at 1.5 million cells/10 cm plate and transfected on the following day with 6 µg of either INF2-GFP or GFP and with either myc-CAP1 or myc-CAP2 as well as dsRed using Lipofectamine 2000 (Thermo Fischer). For each co-immunoprecipitation, 30 µl of Dynabeads™ Protein G (ThermoFischer, 10004D) were used per condition. The beads were washed twice with 600 µl washing buffer (20 mM Tris/HCl pH 8.0, 100 mM NaCl) each time incubating for 5 min on a rotating platform. 2 µg of anti-GFP antibody per condition and 600 µl of washing buffer were added and incubated at room temperature on the rotating platform for 1 h. Subsequently, the beads were washed three times with 500 µl washing buffer. To the beads 30 µl of wash buffer were added. Two days after transfection, HT-22 cells were lysed in a buffer containing 1% Triton X-100 and homogenized. After centrifugation, supernatant was obtained. 600 µl of the supernatant were incubated with the prepared Dynabeads for 2 h at 8 °C. Afterwards, beads were washed three times for five minutes with 500 µl washing buffer at 8 °C. The remaining beads were suspended in 30 µl LB buffer and 30 µl of lysis buffer per condition. For validation, samples were heated for 5 min at 95 °C and applied onto SDS-PAGE and transferred onto an Immobilon-FL membrane. To prove equal loading, total protein staining was performed by using Revert 700 Total Protein Staining according to manufacturer’s protocol and using the Li-COR Odyssey CLx system. Expression levels of myc-CAP constructs, IP was incubated with an antibody against myc bound to a horseradish peroxidase and immunoblots were developed on a Hyperfilm ECL film. For quantifying the myc-CAP constructs we used the ECL method incubating with an anti-myc antibody bound to a horseradish peroxidase, the ECL solution and developing it on a Hyperfilm ECL Film.

### Mass spectrometry

For mass spectrometry, C57BL/6N mice cerebral cortices and hippocampi from 4 animals were used per each developmental time point (E18.5, P10 and P40), making 12 animals in total. Protein lysates from were prepared by addition of 500 µl lysis buffer (0.1 M Tris–HCl, pH 8.0, 0.1 M DTT, 2% SDS) to the tissue and homogenized with 20 strokes using plastic pestle in the 1.5 mL collection tube. Then, lysates were passed through the 30G needle with 1 mL syringe for 5 times and incubated for 5 min at 95 °C. The viscosity of the sample was reduced by sonication with Branson Sonifier 450 (duty cycle 50%, 20 pulses with minimal power settings – output 10–12%) at 4 °C. Non-dissolved cell debris was discarded as a pellet by 10 min centrifugation at 20 °C. Supernatants were treated with 5 mM TCEP for 15 min at 90 °C. After cooling down the sample to room temperature, 10 mM iodoacetamide was added and the sample was incubated for 30 min in the dark. Excess iodoacetamide was neutralized by the addition of excess DTT. Samples were then further processed using a SP3 protocol adapted from previous study [32]. 4 µL of the SP3 bead-slurry was added to 25 µL of each sample (if samples had less volume, volume was adapted to 25 µL by the addition of Millipore water). Subsequently, 29 µL acetonitrile were added. Samples were quickly vortexed and then incubated for 15 min at room temperature. Beads were then separated using a magnetic separator. Supernatant was discarded. Beads were washed two times with 500 µL 70% ethanol. Finally, beads were washed once with 200 µL acetonitrile. Dry beads were incubated over-night with sequencing grade modified trypsin (SERVA) in 100 µL digestion buffer (10% acetonitrile, 50 mM ammoniumbicarbonate) in a thermomixer at 1200 rpm and 30 °C. Beads were separated using a magnetic separator. Supernatant was transferred to new 1.5 mL reaction tubes. 30 µL 2% DMSO were added to the beads and solution was incubated for 5 min in an ultrasonic bath. Subsequently beads were separated using a magnetic separator and supernatant was added to the corresponding tubes containing the supernatants. Subsequently, 30 µL of water were added to the beads, samples were vortexed and spinned down. Magnetic beads were separated again using a magnetic separator and supernatants added to the corresponding supernatants of the previous elution steps. Thereafter, 10 µL of 5% TFA were added to each collection tube. Peptides were then desalted and concentrated using Chromabond C18WP spin columns (Macherey–Nagel, Part No. 730522). Finally, peptides were dissolved in 25 µL of water with 5% acetonitrile and 0.1% formic acid. Mass spectrometric analysis was performed using a timsTOF Pro mass spectrometer (Bruker Daltonic). A nanoElute HPLC system (Bruker Daltonics), equipped with an Aurora column (25 cm × 75 µm) C18 RP column filled with 1.7 µm beads (IonOpticks) was connected online to the mass spectrometer. A portion of approximately 200 ng of peptides was injected directly on the separation column. Sample Loading was performed at a constant pressure of 800 bar.

Separation of the tryptic peptides was achieved at 50 °C column temperature with the following gradient of water/0.1% formic acid (solvent A) and acetonitrile/0.1% formic acid (solvent B) at a flow rate of 400 nL/min: Linear increase from 2%B to 17%B within 60 min, followed by a linear gradient to 25%B within 30 min and linear increase to 37% solvent B in additional 10 min. Finally, B was increased to 95% within 10 min and hold for additional 10 min. The built-in “DDA PASEF-standard_1.1sec_cycletime” method developed by Bruker Daltonics was used for mass spectrometric measurement.

Data analysis was performed using MaxQuant (version 1.6.17.0) with Andromeda search engine against the Uniprot database. Peptides with minimum of seven amino-acid lengths were used and FDR was set to 1% at the peptide and protein level. Protein identification required at least one razor peptide per protein group and label free quantification (LFQ) algorithm was applied. Bioinformatics analysis was performed with Perseus software (1.6.15.0) using LFQ intensity values. Log2 converted LFQ intensity values were normalized by subtraction of the median from the values of individual proteins. Statistical analysis of differential expression was estimated using Student’s t-test (two-sample, unpaired) and corrected for multiple comparison using Benjamini–Hochberg method.

### Statistical analysis

Values are shown as mean values (MV) ± standard error of the means (SEM) based on at least three independent biological replicates, i.e. neurons from three different preparations. Most graphs additionally include values of individual neurons, which are shown in different colors as circles, squares or triangles (Figs. 1F, H-I, 2B-F, I-K, 3B-F, 4E, 5B-F, 6B-F, I-M, S2B-C, S3G-I). Statistical analyses were performed using GraphPad Prism 9. For comparison between two groups, statistical significance was calculated by using Student’s t-test (two-sample, unpaired). For experiments involving more than two groups, statistical significance was tested by performing two-way ANOVA followed by Tukey's multiple comparisons test. Comparison of spine type distribution was tested with χ^2^-test. Table 6 provides detailed information including MV ± SEM, P values, numbers (N) of spines, neurons and independent biological replicates for each spine analysis. Throughout the entire study, experimenters were blinded to the genotype during both image acquisition and analysis.

### Supplementary Information

Below is the link to the electronic supplementary material.

Supplementary file1 Table S1. Complete list of proteins identified by mass spectrometry in hippocampal lysates from E18.5, P10 and P40 C57BL/6N mice (XLSX 1485 KB)

Supplementary file2 Table S2: Complete list of proteins identified by mass spectrometry in cerebral cortex lysates from E18.5, P10 and P40 C57BL/6N mice (XLSX 1584 KB)

Supplementary file3 Table S3: URLs of images showing in situ hybridization data for CAP1, CAP2 and INF2 in the brain, which were extracted from the Allen Mouse Brain Atlas (https://mouse.brain-map.org/) (PDF 9 KB)

Supplementary file4 Table S4. Information about primary antibodies (company, catalogue number, dilution) used for immunocytochemistry and/or immunoblots (PDF 61 KB)

Supplementary file5 Table S5. Information about secondary antibodies (company, catalogue number, dilution) used for immunocytochemistry and/or immunoblots (PDF 13 KB)

Supplementary file6 Table S6A-O: Detailed information on mean values (MV), standard error of the means (SEM), statistical tests, P values as well as numbers (N) of spines, neurons and independent biological replicates for each spine analysis (PDF 250 KB)

Supplementary file7 Table S7. Sequences of CTR-sh and shRNA against either CAP2 or INF2 (PDF 68 KB)

Supplementary file8 Figure S1. (A) This plot supplements mass spectrometry data shown in Fig. 1B and shows the expected increase of CamKII expression and the expected decrease of doublecortin (DCX) expression throughout hippocampal development. Further, it includes expression levels of the house keeping protein glyceraldehyde 3-phosphate dehydrogenase (GAPDH). (B) Expression levels of CAP1, CAP2, INF2, CamKII, DCX and GAPDH determined by mass spectrometry on cerebral cortex lysates from E18.5, P10 and P40 mice. Violin plots to the right show expression level distribution of all proteins in these lysates. Norm. log2 LFQ intensity of zero indicates median expression level of all proteins detected within respective condition. Graph includes values of individual protein samples (N=4), MV±SEM. Statistical comparison of CAP1 and CAP2 expression levels were performed using Student’s t-test and corrected for multiple comparison with Bonferroni method. In situ hybridization and expression color map showing expression of (C) CAP1 and (D) CAP2 in the hippocampus and cerebral cortex from adult mice. Data were extracted from the Allen Mouse Brain Atlas, URLs of the images are provided in Table S3. Immunoblots with antibodies against (E) CAP2 and (F) CAP1 in hippocampal lysates of three adult CAP2-/- mice and CAP2+/+ littermates each. Total protein staining was performed to confirm equal loading. (G) Quantification of CAP1 levels in lysates from CAP2+/+ and CAP2-/- mice normalized to total protein load. Scale bar (mm): 3. ns: P>0.05, ***: P<0.001, ****: P<0.0001 (PDF 2976 KB)

Supplementary file9 Figure S2. (A) Micrographs of GFP-expressing CAP1flx/flx neurons transfected with either Cre-mut and CTR-sh or Cre and CAP2-sh3. Boxes indicate areas shown at higher magnification. Graphs showing (B) spine density and (C) spine volume in neurons transfected with either Cre-mut and CTR-sh or Cre and CAP2-sh. (D) Immunoblots showing CAP2 expression in lysates of cortical neurons upon electroporation of Ctr-sh and three different shRNAs against CAP2. Neurons were co-electroporated with GFP. GFP and β-tubulin were used as loading controls. Red box highlights CAP2-sh3, that has been used for CAP2 knockdown in morphometric analyses (Fig. S2A-C). Scale bar (µm): 20. ***: P<0.001, ****: P<0.0001 (PDF 609 KB)

Supplementary file10 Figure S3. (A) Scheme showing categorization of spine types. Graphs showing (B) length and width of filopodia-like spines, (C) length, head length and head width of thin spines, (D) length and width of stubby spines as well as (E) length, head length and head width of mushroom-like spines in CTR, CAP1-KO, CAP2-KO and dKO neurons. ns: P≥0.05, *: P<0.05 (PDF 1168 KB)

Supplementary file11 Figure S4. Graphs showing (A) length and width of filopodia-like spines, (B) length, head length and head width of thin spines, (C) length and width of stubby spines as well as (D) length, head length and head width of mushroom-like spines in DIV11 CTR and dKO neurons (PDF 1062 KB)

Supplementary file12 Figure S5. (A) In situ hybridization and expression color map showing INF2 expression in the hippocampus, cerebral cortex and striatum from adult mice. Data were extracted from the Allen Mouse Brain Atlas, URL of the image is provided in Table S3. Immunoblots with antibody against GFP in lysates from HT-22 cells expressing (B) myc-CAP1 together with either GFP or INF2-GFP or (C) myc-CAP2 together with either GFP or INF2-GFP. The expression levels of INF2-GFP protein was rather low, but still detectable as a weak band in the input lane. Total protein staining as loading control for both immunoblots is shown in Fig. 5F-G. Scale bar (mm): 1.4 (PDF 1562 KB)

Supplementary file13 Figure S6. (A) Immunoblot showing INF2 expression in HT-22 cells upon expression of CTR-sh or four different shRNA against INF2. GAPDH was used as loading control. Red box highlights INF2-sh3, that has been used for INF2 knockdown in morphometric analyses (Figs. 6A-G, 7H-N). (B) Quantification of INF2 in HT-22 cells expressing CTR-sh or different INF2-sh. N=immunoblots of three independent experiments. Graphs showing (C) length and width of filopodia-like spines, (D) length, head length and head width of thin spines, (E) length and width of stubby spines as well as (F) length, head length and head width of mushroom-like spines in DIV16 CTR neurons expressing either CTR-sh or INF2-sh. (G) Graphs showing (G) length and width of filopodia-like spines, (H) length, head length and head width of thin spines, (I) length and width of stubby spines as well as (J) length, head length and head width of mushroom-like spines in DIV16 CTR neurons expressing either GFP or INF2-GFP. *: P<0.05 (PDF 2447 KB)

Supplementary file14 Figure S7. Graphs showing (A) length and width of filopodia-like spines, (B) length, head length and head width of thin spines, (C) length and width of stubby spines as well as (D) length, head length and head width of mushroom-like spines in DIV16 dKO neurons expressing either GFP or INF2-GFP. (E) Graphs showing (G) length and width of filopodia-like spines, (H) length, head length and head width of thin spines, (I) length and width of stubby spines as well as (J) length, head length and head width of mushroom-like spines in DIV16 dKO neurons expressing either CTR-sh or INF2-sh (PDF 2305 KB)

## Data Availability

The datasets generated during and/or analysed during the current study are available from the corresponding author on reasonable request.

## References

[1] MU A, Fung TS, Francomacaro LM, Huynh T, Kotila T, Svindrych Z, Higgs HN (2020) Regulation of INF2-mediated actin polymerization through site-specific lysine acetylation of actin itself. Proc Natl Acad Sci USA 117:439–447 31871199 10.1073/pnas.1914072117 PMC6955303

[2] A MU, Fung TS, Kettenbach AN, Chakrabarti R, Higgs HN (2019) A complex containing lysine-acetylated actin inhibits the formin INF2. Nat Cell Biol 21:592–602 30962575 10.1038/s41556-019-0307-4 PMC6501848

[3] A MU, Latario CJ, Pickrell LE, Higgs HN (2020) Lysine acetylation of cytoskeletal proteins: Emergence of an actin code. J Cell Biol 219:e202006151 33044556 10.1083/jcb.202006151 PMC7555357

[4] Alimov N, Hoeprich GJ, Padrick SB, Goode BL (2023) Cyclase-associated protein interacts with actin filament barbed ends to promote depolymerization and formin displacement. J Biol Chem 299:105367 37863260 10.1016/j.jbc.2023.105367 PMC10692737

[5] Aspit L, Levitas A, Etzion S, Krymko H, Slanovic L, Zarivach R, Etzion Y, Parvari R (2019) CAP2 mutation leads to impaired actin dynamics and associates with supraventricular tachycardia and dilated cardiomyopathy. J Med Genet 56:228–235 30518548 10.1136/jmedgenet-2018-105498

[6] Bosch M, Castro J, Saneyoshi T, Matsuno H, Sur M, Hayashi Y (2014) Structural and molecular remodeling of dendritic spine substructures during long-term potentiation. Neuron 82:444–459 24742465 10.1016/j.neuron.2014.03.021 PMC4281348

[7] Bosch M, Hayashi Y (2012) Structural plasticity of dendritic spines. Curr Opin Neurobiol 22:383–388 21963169 10.1016/j.conb.2011.09.002 PMC4281347

[8] Boyer O, Nevo F, Plaisier E, Funalot B, Gribouval O, Benoit G, Huynh Cong E, Arrondel C, Tete MJ, Montjean R, Richard L, Karras A, Pouteil-Noble C, Balafrej L, Bonnardeaux A, Canaud G, Charasse C, Dantal J, Deschenes G, Deteix P, Dubourg O, Petiot P, Pouthier D, Leguern E, Guiochon-Mantel A, Broutin I, Gubler MC, Saunier S, Ronco P, Vallat JM, Alonso MA, Antignac C, Mollet G (2011) INF2 mutations in Charcot-Marie-Tooth disease with glomerulopathy. N Engl J Med 365:2377–2388 22187985 10.1056/NEJMoa1109122

[9] Breitsprecher D, Goode BL (2013) Formins at a glance. J Cell Sci 126:1–7 23516326 10.1242/jcs.107250 PMC3603506

[10] Brown EJ, Schlondorff JS, Becker DJ, Tsukaguchi H, Tonna SJ, Uscinski AL, Higgs HN, Henderson JM, Pollak MR (2010) Mutations in the formin gene INF2 cause focal segmental glomerulosclerosis. Nat Genet 42:72–76 20023659 10.1038/ng.505 PMC2980844

[11] Calabrese B, Jones SL, Shiraishi-Yamaguchi Y, Lingelbach M, Manor U, Svitkina TM, Higgs HN, Shih AY, Halpain S (2022) INF2-mediated actin filament reorganization confers intrinsic resilience to neuronal ischemic injury. Nat Commun 13:6037 36229429 10.1038/s41467-022-33268-y PMC9558009

[12] Cheema H, Bertoli-Avella AM, Skrahina V, Anjum MN, Waheed N, Saeed A, Beetz C, Perez-Lopez J, Rocha ME, Alawbathani S, Pereira C, Hovakimyan M, Patric IRP, Paknia O, Ameziane N, Cozma C, Bauer P, Rolfs A (2020) Genomic testing in 1019 individuals from 349 Pakistani families results in high diagnostic yield and clinical utility. NPJ Genom Med 5:44 33083013 10.1038/s41525-020-00150-z PMC7536406

[13] Colpan M, Iwanski J, Gregorio CC (2021) CAP2 is a regulator of actin pointed end dynamics and myofibrillogenesis in cardiac muscle. Commun Biol 4:365 33742108 10.1038/s42003-021-01893-w PMC7979805

[14] Dominguez R (2020) Actin: Post-translational Modification of Actin Linked to Formin Inhibition. Curr Biol 29:R367–R370 10.1016/j.cub.2019.03.061 31112687

[15] Drazic A, Aksnes H, Marie M, Boczkowska M, Varland S, Timmerman E, Foyn H, Glomnes N, Rebowski G, Impens F, Gevaert K, Dominguez R, Arnesen T (2018) NAA80 is actin’s N-terminal acetyltransferase and regulates cytoskeleton assembly and cell motility. Proc Natl Acad Sci USA 115:4399–4404 29581253 10.1073/pnas.1718336115 PMC5924898

[16] Duffney LJ, Zhong P, Wei J, Matas E, Cheng J, Qin L, Ma K, Dietz DM, Kajiwara Y, Buxbaum JD, Yan Z (2015) Autism-like Deficits in Shank3-Deficient Mice Are Rescued by Targeting Actin Regulators. Cell Rep 11:1400–1413 26027926 10.1016/j.celrep.2015.04.064 PMC4464902

[17] Durand CM, Perroy J, Loll F, Perrais D, Fagni L, Bourgeron T, Montcouquiol M, Sans N (2012) SHANK3 mutations identified in autism lead to modification of dendritic spine morphology via an actin-dependent mechanism. Mol Psychiatry 17:71–84 21606927 10.1038/mp.2011.57 PMC3252613

[18] Field J, Ye DZ, Shinde M, Liu F, Schillinger KJ, Lu M, Wang T, Skettini M, Xiong Y, Brice AK, Chung DC, Patel VV (2015) CAP2 in cardiac conduction, sudden cardiac death and eye development. Sci Rep 5:17256 26616005 10.1038/srep17256 PMC4663486

[19] Fromer M, Pocklington AJ, Kavanagh DH, Williams HJ, Dwyer S, Gormley P, Georgieva L, Rees E, Palta P, Ruderfer DM, Carrera N, Humphreys I, Johnson JS, Roussos P, Barker DD, Banks E, Milanova V, Grant SG, Hannon E, Rose SA, Chambert K, Mahajan M, Scolnick EM, Moran JL, Kirov G, Palotie A, McCarroll SA, Holmans P, Sklar P, Owen MJ, Purcell SM, O’Donovan MC (2014) De novo mutations in schizophrenia implicate synaptic networks. Nature 506:179–184 24463507 10.1038/nature12929 PMC4237002

[20] Gilman SR, Iossifov I, Levy D, Ronemus M, Wigler M, Vitkup D (2011) Rare de novo variants associated with autism implicate a large functional network of genes involved in formation and function of synapses. Neuron 70:898–907 21658583 10.1016/j.neuron.2011.05.021 PMC3607702

[21] Görlich A, Zimmermann AM, Schober D, Bottcher RT, Sassoe-Pognetto M, Friauf E, Witke W, Rust MB (2012) Preserved morphology and physiology of excitatory synapses in profilin1-deficient mice. PLoS ONE 7:e30068 22253883 10.1371/journal.pone.0030068 PMC3256187

[22] Goodson M, Rust MB, Witke W, Bannerman D, Mott R, Ponting CP, Flint J (2012) Cofilin-1: a modulator of anxiety in mice. PLoS Genet 8:e1002970 23055942 10.1371/journal.pgen.1002970 PMC3464202

[23] Gu J, Lee CW, Fan Y, Komlos D, Tang X, Sun C, Yu K, Hartzell HC, Chen G, Bamburg JR, Zheng JQ (2010) ADF/cofilin-mediated actin dynamics regulate AMPA receptor trafficking during synaptic plasticity. Nat Neurosci 13:1208–1215 20835250 10.1038/nn.2634 PMC2947576

[24] Gurunathan S, Sebastian J, Baker J, Abdel-Hamid HZ, West SC, Feingold B, Peche V, Reyes-Mugica M, Madan-Khetarpal S, Field J (2021) A homozygous CAP2 pathogenic variant in a neonate presenting with rapidly progressive cardiomyopathy and nemaline rods. Am J Med Genet A 188:970–977 34862840 10.1002/ajmg.a.62590

[25] Heinze A, Rust MB (2023) Loss of the actin regulator cyclase-associated protein 1 (CAP1) modestly affects dendritic spine remodeling during synaptic plasticity. Eur J Cell Biol 102:151357 37634312 10.1016/j.ejcb.2023.151357

[26] Heinze A, Schuldt C, Khudayberdiev S, van Bommel B, Hacker D, Schulz TG, Stringhi R, Marcello E, Mikhaylova M, Rust MB (2022) Functional interdependence of the actin regulators CAP1 and cofilin1 in control of dendritic spine morphology. Cell Mol Life Sci 79:558 36264429 10.1007/s00018-022-04593-8 PMC9585016

[27] Hering H, Sheng M (2001) Dendritic spines: structure, dynamics and regulation. Nat Rev Neurosci 2:880–888 11733795 10.1038/35104061

[28] Hlushchenko I, Koskinen M, Hotulainen P (2016) Dendritic spine actin dynamics in neuronal maturation and synaptic plasticity. Cytoskeleton (Hoboken) 73:435–441 26849484 10.1002/cm.21280

[29] Holtmaat A, Svoboda K (2009) Experience-dependent structural synaptic plasticity in the mammalian brain. Nat Rev Neurosci 10:647–658 19693029 10.1038/nrn2699

[30] Hotulainen P, Hoogenraad CC (2010) Actin in dendritic spines: connecting dynamics to function. J Cell Biol 189:619–629 20457765 10.1083/jcb.201003008 PMC2872912

[31] Hotulainen P, Llano O, Smirnov S, Tanhuanpaa K, Faix J, Rivera C, Lappalainen P (2009) Defining mechanisms of actin polymerization and depolymerization during dendritic spine morphogenesis. J Cell Biol 185:323–339 19380880 10.1083/jcb.200809046 PMC2700375

[32] Hughes CS, Foehr S, Garfield DA, Furlong EE, Steinmetz LM, Krijgsveld J (2014) Ultrasensitive proteome analysis using paramagnetic bead technology. Mol Syst Biol 10:757 25358341 10.15252/msb.20145625 PMC4299378

[33] Johnston AB, Collins A, Goode BL (2015) High-speed depolymerization at actin filament ends jointly catalysed by Twinfilin and Srv2/CAP. Nat Cell Biol 17:1504–1511 26458246 10.1038/ncb3252 PMC4808055

[34] Kepser LJ, Damar F, De Cicco T, Chaponnier C, Proszynski TJ, Pagenstecher A, Rust MB (2019) CAP2 deficiency delays myofibril actin cytoskeleton differentiation and disturbs skeletal muscle architecture and function. Proc Natl Acad Sci USA 116:8397–8402 30962377 10.1073/pnas.1813351116 PMC6486752

[35] Khudayberdiev S, Weiss K, Heinze A, Colombaretti D, Trausch N, Linne U, Rust MB (2024) The actin-binding protein CAP1 represses MRTF-SRF-dependent gene expression in mouse cerebral cortex. Sci Signal. 17:eadj0032 38713765 10.1126/scisignal.adj0032

[36] Kotila T, Kogan K, Enkavi G, Guo S, Vattulainen I, Goode BL, Lappalainen P (2018) Structural basis of actin monomer re-charging by cyclase-associated protein. Nat Commun 9:1892 29760438 10.1038/s41467-018-04231-7 PMC5951797

[37] Kotila T, Wioland H, Enkavi G, Kogan K, Vattulainen I, Jegou A, Romet-Lemonne G, Lappalainen P (2019) Mechanism of synergistic actin filament pointed end depolymerization by cyclase-associated protein and cofilin. Nat Commun 10:5320 31757941 10.1038/s41467-019-13213-2 PMC6876575

[38] Lamprecht R, LeDoux J (2004) Structural plasticity and memory. Nat Rev Neurosci 5:45–54 14708003 10.1038/nrn1301

[39] Lei W, Omotade OF, Myers KR, Zheng JQ (2016) Actin cytoskeleton in dendritic spine development and plasticity. Curr Opin Neurobiol 39:86–92 27138585 10.1016/j.conb.2016.04.010 PMC4987222

[40] Lein ES, Hawrylycz MJ, Ao N, Ayres M, Bensinger A, Bernard A, Boe AF, Boguski MS, Brockway KS, Byrnes EJ, Chen L, Chen L, Chen TM, Chin MC, Chong J, Crook BE, Czaplinska A, Dang CN, Datta S, Dee NR, Desaki AL, Desta T, Diep E, Dolbeare TA, Donelan MJ, Dong HW, Dougherty JG, Duncan BJ, Ebbert AJ, Eichele G, Estin LK, Faber C, Facer BA, Fields R, Fischer SR, Fliss TP, Frensley C, Gates SN, Glattfelder KJ, Halverson KR, Hart MR, Hohmann JG, Howell MP, Jeung DP, Johnson RA, Karr PT, Kawal R, Kidney JM, Knapik RH, Kuan CL, Lake JH, Laramee AR, Larsen KD, Lau C, Lemon TA, Liang AJ, Liu Y, Luong LT, Michaels J, Morgan JJ, Morgan RJ, Mortrud MT, Mosqueda NF, Ng LL, Ng R, Orta GJ, Overly CC, Pak TH, Parry SE, Pathak SD, Pearson OC, Puchalski RB, Riley ZL, Rockett HR, Rowland SA, Royall JJ, Ruiz MJ, Sarno NR, Schaffnit K, Shapovalova NV, Sivisay T, Slaughterbeck CR, Smith SC, Smith KA, Smith BI, Sodt AJ, Stewart NN, Stumpf KR, Sunkin SM, Sutram M, Tam A, Teemer CD, Thaller C, Thompson CL, Varnam LR, Visel A, Whitlock RM, Wohnoutka PE, Wolkey CK, Wong VY et al (2007) Genome-wide atlas of gene expression in the adult mouse brain. Nature 445:168–176 17151600 10.1038/nature05453

[41] Lin WH, Nebhan CA, Anderson BR, Webb DJ (2010) Vasodilator-stimulated phosphoprotein (VASP) induces actin assembly in dendritic spines to promote their development and potentiate synaptic strength. J Biol Chem 285:36010–36020 20826790 10.1074/jbc.M110.129841 PMC2975223

[42] Minegishi T, Kastian RF, Inagaki N (2023) Mechanical regulation of synapse formation and plasticity. Semin Cell Dev Biol 140:82–89 35659473 10.1016/j.semcdb.2022.05.017

[43] Muffels IJJ, Wiame E, Fuchs SA, Massink MPG, Rehmann H, Musch JLI, Van Haaften G, Vertommen D, van Schaftingen E, van Hasselt PM (2021) NAA80 bi-allelic missense variants result in high-frequency hearing loss, muscle weakness and developmental delay. Brain Commun 3:fcab256 34805998 10.1093/braincomms/fcab256 PMC8599064

[44] Okamoto K, Nagai T, Miyawaki A, Hayashi Y (2004) Rapid and persistent modulation of actin dynamics regulates postsynaptic reorganization underlying bidirectional plasticity. Nat Neurosci 7:1104–1112 15361876 10.1038/nn1311

[45] Peche VS, Holak TA, Burgute BD, Kosmas K, Kale SP, Wunderlich FT, Elhamine F, Stehle R, Pfitzer G, Nohroudi K, Addicks K, Stockigt F, Schrickel JW, Gallinger J, Schleicher M, Noegel AA (2012) Ablation of cyclase-associated protein 2 (CAP2) leads to cardiomyopathy. Cell Mol Life Sci 70:527–543 22945801 10.1007/s00018-012-1142-y PMC11113306

[46] Pelucchi S, Vandermeulen L, Pizzamiglio L, Aksan B, Yan J, Konietzny A, Bonomi E, Borroni B, Padovani A, Rust MB, Di Marino D, Mikhaylova M, Mauceri D, Antonucci F, Edefonti V, Gardoni F, Di Luca M, Marcello E (2020) CAP2 dimerization regulates cofilin in synaptic plasticity and Alzheimer’s disease. Brain Commun. 2:fcaa086 33094279 10.1093/braincomms/fcaa086 PMC7566557

[47] Phillips M, Pozzo-Miller L (2015) Dendritic spine dysgenesis in autism related disorders. Neurosci Lett 601:30–40 25578949 10.1016/j.neulet.2015.01.011 PMC4496332

[48] Pyronneau A, He Q, Hwang JY, Porch M, Contractor A, Zukin RS (2017) Aberrant Rac1-cofilin signaling mediates defects in dendritic spines, synaptic function, and sensory perception in fragile X syndrome. Sci Signal 10:eaan0852 29114038 10.1126/scisignal.aan0852 PMC5988355

[49] Quach TT, Stratton HJ, Khanna R, Kolattukudy PE, Honnorat J, Meyer K, Duchemin AM (2021) Intellectual disability: dendritic anomalies and emerging genetic perspectives. Acta Neuropathol 141:139–158 33226471 10.1007/s00401-020-02244-5 PMC7855540

[50] Rotty JD, Wu C, Bear JE (2013) New insights into the regulation and cellular functions of the ARP2/3 complex. Nat Rev Mol Cell Biol 14:7–12 23212475 10.1038/nrm3492

[51] Rust MB (2015) ADF/cofilin: a crucial regulator of synapse physiology and behavior. Cell Mol Life Sci 72:3521–3529 26037722 10.1007/s00018-015-1941-z PMC11113150

[52] Rust MB (2015) Novel functions for ADF/cofilin in excitatory synapses - lessons from gene-targeted mice. Commun Integr Biol 8:e1114194 27066177 10.1080/19420889.2015.1114194 PMC4802768

[53] Rust MB, Gurniak CB, Renner M, Vara H, Morando L, Gorlich A, Sassoe-Pognetto M, Banchaabouchi MA, Giustetto M, Triller A, Choquet D, Witke W (2010) Learning, AMPA receptor mobility and synaptic plasticity depend on n-cofilin-mediated actin dynamics. EMBO J 29:1889–1902 20407421 10.1038/emboj.2010.72 PMC2885936

[54] Rust MB, Khudayberdiev S, Pelucchi S, Marcello E (2020) CAPt’n of actin dynamics: recent advances in the molecular, developmental and physiological functions of cyclase-associated protein (CAP). Front Cell Dev Biol 8:586631 33072768 10.3389/fcell.2020.586631 PMC7543520

[55] Rust MB, Marcello E (2022) Disease association of cyclase-associated protein (CAP): lessons from gene-targeted mice and human genetic studies. Eur J Cell Biol 101:151207 35150966 10.1016/j.ejcb.2022.151207

[56] Rust MB, Maritzen T (2015) Relevance of presynaptic actin dynamics for synapse function and mouse behavior. Exp Cell Res 335:165–171 25579398 10.1016/j.yexcr.2014.12.020

[57] Schneider F, Duong TA, Metz I, Winkelmeier J, Hubner CA, Endesfelder U, Rust MB (2021) Mutual functional dependence of cyclase-associated protein 1 (CAP1) and cofilin1 in neuronal actin dynamics and growth cone function. Prog Neurobiol 202:102050 33845164 10.1016/j.pneurobio.2021.102050

[58] Schneider F, Metz I, Khudayberdiev S, Rust MB (2021) Functional redundancy of cyclase-associated proteins CAP1 and CAP2 in differentiating neurons. Cells 10:1525 34204261 10.3390/cells10061525 PMC8234816

[59] Schneider F, Duong TA, Rust MB (2021c) Neuron replating – a powerful and versatile approach to study early aspects of neuron differentiation. eNeuro. 8:ENEURO.0536–20.2021 10.1523/ENEURO.0536-20.2021 PMC8143016 33958372

[60] Schratt GM, Tuebing F, Nigh EA, Kane CG, Sabatini ME, Kiebler M, Greenberg ME (2006) A brain-specific microRNA regulates dendritic spine development. Nature 439:283–289 16421561 10.1038/nature04367

[61] Shekhar S, Chung J, Kondev J, Gelles J, Goode BL (2019) Synergy between Cyclase-associated protein and Cofilin accelerates actin filament depolymerization by two orders of magnitude. Nat Commun 10:5319 31757952 10.1038/s41467-019-13268-1 PMC6876572

[62] Sheng M, Hoogenraad CC (2007) The postsynaptic architecture of excitatory synapses: a more quantitative view. Annu Rev Biochem 76:823–847 17243894 10.1146/annurev.biochem.76.060805.160029

[63] Spence EF, Kanak DJ, Carlson BR, Soderling SH (2016) The Arp2/3 complex is essential for distinct stages of spine synapse maturation, including synapse unsilencing. J Neurosci 36:9696–9709 27629719 10.1523/JNEUROSCI.0876-16.2016 PMC5039249

[64] Spence EF, Soderling SH (2015) Actin out: regulation of the synaptic cytoskeleton. J Biol Chem 290:28613–28622 26453304 10.1074/jbc.R115.655118 PMC4661376

[65] Sungur AÖ, Stemmler L, Wöhr M, Rust MB (2018) Impaired object recognition but normal social behavior and ultrasonic communication in cofilin1 mutant mice. Front Behav Neurosci 12:25 29515378 10.3389/fnbeh.2018.00025 PMC5825895

[66] Towsif EM, Shekhar S (2023) Cyclase-associated protein is a pro-formin anti-capping processive depolymerase of actin barbed and pointed ends. *bioRxiv*. 2023 Dec 01: 2023.11.30.569482

[67] Varland S, Vandekerckhove J, Drazic A (2019) Actin post-translational modifications: the cinderella of cytoskeletal control. Trends Biochem Sci 44:502–516 30611609 10.1016/j.tibs.2018.11.010

[68] Wolf M, Zimmermann AM, Gorlich A, Gurniak CB, Sassoe-Pognetto M, Friauf E, Witke W, Rust MB (2015) ADF/cofilin controls synaptic actin dynamics and regulates synaptic vesicle mobilization and exocytosis. Cereb Cortex 25:2863–2875 24770705 10.1093/cercor/bhu081

[69] Xiong Y, Bedi K, Berritt S, Attipoe BK, Brooks TG, Wang K, Margulies KB, Field J (2019) Targeting MRTF/SRF in CAP2-dependent dilated cardiomyopathy delays disease onset. JCI Insight 4:e124629 30762586 10.1172/jci.insight.124629 PMC6483011

[70] Yang Y, Liu JJ (2022) Structural LTP: signal transduction, actin cytoskeleton reorganization, and membrane remodeling of dendritic spines. Curr Opin Neurobiol 74:102534 35398661 10.1016/j.conb.2022.102534

[71] Yuste R, Bonhoeffer T (2001) Morphological changes in dendritic spines associated with long-term synaptic plasticity. Annu Rev Neurosci 24:1071–1089 11520928 10.1146/annurev.neuro.24.1.1071

[72] Yuste R, Bonhoeffer T (2004) Genesis of dendritic spines: insights from ultrastructural and imaging studies. Nat Rev Neurosci 5:24–34 14708001 10.1038/nrn1300

[73] Zhang Z, Ye M, Li Q, You Y, Yu H, Ma Y, Mei L, Sun X, Wang L, Yue W, Li R, Li J, Zhang D (2019) The schizophrenia susceptibility gene OPCML regulates spine maturation and cognitive behaviors through Eph-Cofilin signaling. Cell Rep 29(49–61):e47 10.1016/j.celrep.2019.08.091 31577955

[74] Zimmermann AM, Jene T, Wolf M, Gorlich A, Gurniak CB, Sassoe-Pognetto M, Witke W, Friauf E, Rust MB (2015) Attention-deficit/hyperactivity disorder-like phenotype in a mouse model with impaired actin dynamics. Biol Psychiatry 78:95–106 24768258 10.1016/j.biopsych.2014.03.011

[75] Zuo Y, Lin A, Chang P, Gan WB (2005) Development of long-term dendritic spine stability in diverse regions of cerebral cortex. Neuron 46:181–189 15848798 10.1016/j.neuron.2005.04.001

