# Supplementary material for: Cyclase-associated protein (CAP) inhibits inverted formin 2 (INF2) to induce dendritic spine maturation
Source: Cell Mol Life Sci. 2024 Aug 18;81(1):353. doi: 10.1007/s00018-024-05393-y (PMC11335277; doi:10.1007/s00018-024-05393-y)
Supplement: Supplementary file 3 — Supplementary file3 Table S3: URLs of images showing in situ hybridization data for CAP1, CAP2 and INF2 in the brain, which were extracted from the Allen Mouse Brain Atlas (https://mouse.brain-map.org/) (PDF 9 KB) [file 18_2024_5393_MOESM3_ESM.pdf]

Table S3: URLs for images extracted from Allen Mouse Brain Atlas

Gen	URL
CAP1	https://mouse.brain-map.org/experiment/siv?id=323396&imageId=235676&initImage=expression&colormap=0.5,1,0,256,4&coordSystem=pixel&x=5312.5&y=3608.5&z=1
CAP2	https://mouse.brain-map.org/experiment/siv?id=70436737&imageId=70142663&initImage=ish&coordSystem=pixel&x=5304.5&y=3728.5&z=1
INF2	https://mouse.brain-map.org/experiment/siv?id=69202902&imageId=69132812&initImage=expression&colormap=0.5,1,0,256,4&coordSystem=pixel&x=7112.5&y=3608.5&z=1
