# Supplementary material for: Cyclase-associated protein (CAP) inhibits inverted formin 2 (INF2) to induce dendritic spine maturation
Source: Cell Mol Life Sci. 2024 Aug 18;81(1):353. doi: 10.1007/s00018-024-05393-y (PMC11335277; doi:10.1007/s00018-024-05393-y)
Supplement: Supplementary file 4 — Supplementary file4 Table S4. Information about primary antibodies (company, catalogue number, dilution) used for immunocytochemistry and/or immunoblots (PDF 61 KB) [file 18_2024_5393_MOESM4_ESM.pdf]

Table S4: List of primary antibodies used for immunocytochemistry (ICC) and immunoblots (IB)

		Dilution			
**Antibody**	**Host**	**ICC**	**IB**	**Supplier**	**Ordering number**
anti-GFP	rabbit	1:1,000	1:1,000	Thermo Fisher Scientific	G10362
anti-c-myc	mouse	1:200		Thermo Fisher Scientific	13-2500
anti-CAP1	mouse	1:200	1:1,000	Abnova	H00010487-M02
anti-CAP2	rabbit	1:200	1:1,000	Proteintech	15865.1-AP
anti-INF2	rabbit	1:50	1:2,000	Millipore	ABT61
anti-GAPDH	mouse		1:1,000	R&D System	MAB5718
anti-ß-tubulin	mouse		1:2,000	Millipore	MAB1637
anti-synaptophysin	rabbit		1:2,000	Synaptic Systems	101002
anti-PSD-95	mouse		1:2,000	Thermo Fisher Scientific	MA1-046
anti-β-actin	mouse		1:4,000	Synaptic Sytems	251011
