# Supplementary material for: Cyclase-associated protein (CAP) inhibits inverted formin 2 (INF2) to induce dendritic spine maturation
Source: Cell Mol Life Sci. 2024 Aug 18;81(1):353. doi: 10.1007/s00018-024-05393-y (PMC11335277; doi:10.1007/s00018-024-05393-y)
Supplement: Supplementary file 5 — Supplementary file5 Table S5. Information about secondary antibodies (company, catalogue number, dilution) used for immunocytochemistry and/or immunoblots (PDF 13 KB) [file 18_2024_5393_MOESM5_ESM.pdf]

Table S5: List of secondary antibodies used for immunocytochemistry (ICC) and immunoblots (IB)

		Dilution			
Antibody	Host	ICC	IB	Supplier	Ordering number
anti-rabbit-AlexaFluor488	goat	1:2,000		Thermo Fisher Scientific	A-11034
anti-mouse-AlexaFluor647	donkey	1:2,000		Thermo Fisher Scientific	A-31571
anti-rabbit-AlexaFluor546	goat	1:2,000		Thermo Fisher Scientific	A-11035
anti-rabbit-HRP	goat		1:20,000	Thermo Fisher Scientific	31460
anti-mouse-HRP	goat		1:20,000	Thermo Fisher Scientific	31430
