# Supplementary material for: Cyclase-associated protein (CAP) inhibits inverted formin 2 (INF2) to induce dendritic spine maturation
Source: Cell Mol Life Sci. 2024 Aug 18;81(1):353. doi: 10.1007/s00018-024-05393-y (PMC11335277; doi:10.1007/s00018-024-05393-y)
Supplement: Supplementary file 6 — Supplementary file6 Table S6A-O: Detailed information on mean values (MV), standard error of the means (SEM), statistical tests, P values as well as numbers (N) of spines, neurons and independent biological replicates for each spine analysis (PDF 250 KB) [file 18_2024_5393_MOESM6_ESM.pdf]

Table S6A-O. Detailed information on MV±SEM, P values, numbers (N) of spines, neurons, independent biological replicates for all spine analysis.

Table S6A. Data shown in Fig. 1H-I

Group	MV±SEM	ANOVA	Tukey's multiple comparisons test	P	
Spine density (μm^{-1})					
$CAP2^{+/+}$	0.489±0.017	P=0.576	$CAP2^{+/+}$ vs. $CAP2^{+/-}$	0.733	ns
$CAP2^{+/-}$	0.484±0.018		$CAP2^{+/+}$ vs. $CAP2^{-/-}$	0.961	ns
$CAP2^{-/-}$	0.498±0.022		$CAP2^{+/-}$ vs. $CAP2^{-/-}$	0.570	ns
Spine volume (a.u.)					
$CAP2^{+/+}$	0.185±0.006	P=0.979	$CAP2^{+/+}$ vs. $CAP2^{+/-}$	0.985	ns
$CAP2^{+/-}$	0.181±0.005		$CAP2^{+/+}$ vs. $CAP2^{-/-}$	0.999	ns
$CAP2^{-/-}$	0.179±0.005		$CAP2^{+/-}$ vs. $CAP2^{-/-}$	0.980	ns

N≥250 spines per neuron, 9 neurons per group and experiment, 3 independent experiments. ns: P≥0.05.

Table S6B. Data shown in Fig. 2B-F

Group	MV±SEM	Anova	Tukey's multiple comparisons test	P	
Spine density (μm^{-1})					
CTR	0.399±0.017	P<0.0001	CTR vs. CAP1-KO	0.1668	ns
CAP1-KO	0.348±0.021		CTR vs. CAP2-KO	0.3667	ns
CAP2-KO	0.359±0.017		CTR vs. dKO	<0.0001	****
dKO	0.243±0.013		CAP1-KO vs. CAP2-KO	0.9675	ns
			CAP1-KO vs. dKO	0.0003	***
			CAP2-KO vs. dKO	<0.0001	****
Spine volume (a.u.)					
CTR	0.238±0.013	P<0.0001	CTR vs. CAP1-KO	0.0408	*
CAP1-KO	0.296±0.022		CTR vs. CAP2-KO	>0.9999	ns
CAP2-KO	0.237±0.016		CTR vs. dKO	0.0440	*
dKO	0.179±0.009		CAP1-KO vs. CAP2-KO	0.0347	*
			CAP1-KO vs. dKO	<0.0001	****
			CAP2-KO vs. dKO	0.0514	ns
Spine length (µm)					
CTR	1.316±0.078	P=0.0010	CTR vs. CAP1-KO	0.3064	ns
CAP1-KO	1.517±0.059		CTR vs. CAP2-KO	0.9907	ns
CAP2-KO	1.281±0.047		CTR vs. dKO	0.0049	**
dKO	1.718±0.114		CAP1-KO vs. CAP2-KO	0.1819	ns
			CAP1-KO vs. dKO	0.3091	ns
			CAP2-KO vs. dKO	0.0020	**
Head length (µm)					
CTR	0.937±0.056	P<0.0001	CTR vs. CAP1-KO	>0.9999	ns
CAP1-KO	0.941±0.038		CTR vs. CAP2-KO	0.3726	ns
CAP2-KO	0.810±0.042		CTR vs. dKO	0.0033	**
dKO	1.223±0.071		CAP1-KO vs. CAP2-KO	0.3500	ns
			CAP1-KO vs. dKO	0.0038	**
			CAP2-KO vs. dKO	<0.0001	****
Head width (µm)					

CTR	0.530±0.018	P<0.0001	CTR vs. CAP1-KO	0.0036	**
CAP1-KO	0.689±0.040		CTR vs. CAP2-KO	0.2278	ns
CAP2-KO	0.615±0.028		CTR vs. dKO	0.3541	ns
dKO	0.456±0.030		CAP1-KO vs. CAP2-KO	0.3453	ns
			CAP1-KO vs. dKO	<0.0001	****
			CAP2-KO vs. dKO	0.0038	**

Spine volume and density: N≥200 spines per neuron, 5 neurons per group and experiment, 3 independent experiments. Spine morphology: N≥15 spines per neuron, 5 neurons per group and experiment, 3 independent experiments. ns: P≥0.05, *: P<0.05, **: P<0.01, ***: P<0.001, ****: P<0.0001.

Table S6C. Data shown in Fig. 3C-H

Group	MV±SEM	Anova	Tukey's multiple comparisons test	P	
	**Spine length (µm)**				
CTR	1.678±0.088	P=0.2402	CTR vs. CAP1-GFP	0.653	ns
+ CAP1-GFP	1.456±0.059		CTR vs. CAP2-GFP	0.812	ns
+ CAP2-GFP	1.483±0.065		CTR vs. CAP1-GFP + CAP2-GFP	0.898	ns
+ CAP1-GFP/ CAP2-GFP	1.635±0.040		CAP1-GFP vs. CAP2-GFP	0.993	ns
			CAP1-GFP vs. CAP1-GFP + CAP2-GFP	0.258	ns
			CAP2-GFP vs. CAP1-GFP + CAP2-GFP	0.396	ns
	**Head length (µm)**				
CTR	1.006±0.069	P=0.667	CTR vs. CAP1-GFP	0.775	ns
+ CAP1-GFP	0.935±0.037		CTR vs. CAP2-GFP	0.880	ns
+ CAP2-GFP	0.951±0.043		CTR vs. CAP1-GFP + CAP2-GFP	0.999	ns
+ CAP1-GFP/ CAP2-GFP	1.008±0.049		CAP1-GFP vs. CAP2-GFP	0.997	ns
			CAP1-GFP vs. CAP1-GFP + CAP2-GFP	0.761	ns
			CAP2-GFP vs. CAP1-GFP + CAP2-GFP	0.869	ns
	**Head width (µm)**				
CTR	0.633±0.015	P=0.0388	CTR vs. CAP1-GFP	0.889	ns
+ CAP1-GFP	0.614±0.020		CTR vs. CAP2-GFP	0.228	ns
+ CAP2-GFP	0.687±0.021		CTR vs. CAP1-GFP + CAP2-GFP	0.512	ns
+ CAP1-GFP/ CAP2-GFP	0.672±0.019		CAP1-GFP vs. CAP2-GFP	0.049	*
			CAP1-GFP vs. CAP1-GFP + CAP2-GFP	0.163	ns
			CAP2-GFP vs. CAP1-GFP + CAP2-GFP	0.949	ns

Group	MV±SEM	Anova	Tukey's multiple comparisons test	P	
**Spine length (µm)**					
dKO	2.012±0.091	P<0.0001	dKO vs. CAP1-GFP	<0.0001	****
+ CAP1-GFP	1.441±0.049		dKO vs. CAP2-GFP	<0.0001	****
+ CAP2-GFP	1.424±0.079		dKO vs. CAP1-GFP + CAP2-GFP	0.0013	**
+ CAP1-GFP/ CAP2-GFP	1.608±0.052		CAP1-GFP vs. CAP2-GFP	0.9984	ns
			CAP1-GFP vs. CAP1-GFP + CAP2-GFP	0.3710	ns
			CAP2-GFP vs. CAP1-GFP + CAP2-GFP	0.2878	ns
**Head length (µm)**					
dKO	1.521±0.101	P<0.0001	dKO vs. CAP1-GFP	0.0002	***
+ CAP1-GFP	1.038±0.059		dKO vs. CAP2-GFP	<0.0001	****
+ CAP2-GFP	0.998±0.080		dKO vs. CAP1-GFP + CAP2-GFP	0.0001	***
+ CAP1-GFP/	1.018±0.039		CAP1-GFP vs. CAP2-GFP	0.982	ns

CAP2-GFP			CAP1-GFP vs. CAP1-GFP + CAP2-GFP<br>CAP2-GFP vs. CAP1-GFP + CAP2-GFP	0.998<br>0.998	ns<br>ns
			**Head width (µm)**		
dKO<br>+ CAP1-GFP<br>+ CAP2-GFP<br>+ CAP1-GFP/<br>CAP2-GFP	0.460±0.018<br>0.569±0.026<br>0.601±0.030<br>0.581±0.028	P=0.0023	dKO vs. CAP1-GFP<br>dKO vs. CAP2-GFP<br>dKO vs. CAP1-GFP + CAP2-GFP<br>CAP1-GFP vs. CAP2-GFP<br>CAP1-GFP vs. CAP1-GFP + CAP2-GFP<br>CAP2-GFP vs. CAP1-GFP + CAP2-GFP	0.0309<br>0.0029<br>0.0128<br>0.8373<br>0.9876<br>0.9571	*<br>**<br>*<br>ns<br>ns<br>ns

N≥15 spines per neuron, 5 neurons per group and experiment, 3 independent experiments. ns: P≥0.05, *: P<0.05.

Group	MV±SEM	Anova	Tukey's multiple comparisons test	P	
			**Spine length (µm)**		
CTR<br>vs dKO		P=0.2503	CTR vs. dKO<br>CTR vs. dKO CAP1-GFP<br>CTR vs. dKO CAP2-GFP<br>CTR vs dKO CAP1-GFP + CAP2 GFP	0.0009<br>0.7498<br>0.6545<br>0.9954	***<br>ns<br>ns<br>ns
			**Head length (µm)**		
CTR<br>vs dKO		P=0.419	CTR vs. dKO<br>CTR vs. dKO CAP1-GFP<br>CTR vs. dKO CAP2-GFP<br>CTR vs dKO CAP1-GFP + CAP2 GFP	>0.001<br>0.998<br>0.999<br>0.999	***<br>ns<br>ns<br>ns
			**Head width (µm)**		
CTR<br>vs dKO		P=0.031	CTR vs. dKO<br>CTR vs. dKO CAP1-GFP<br>CTR vs. dKO CAP2-GFP<br>CTR vs dKO CAP1-GFP + CAP2 GFP	>0.0001<br>0.273<br>0.842<br>0.487	****<br>ns<br>ns<br>*

N≥15 spines per neuron, 5 neurons per group and experiment, 3 independent experiments. ns: P≥0.05, *: P<0.05, ***: P<0.001, ****: P<0.0001.

Table S6D. Data shown in Fig. 4B-F

	CTR (DIV11)	dKO (DIV11)	P	
Spine density (μm^{-1})	0.36±0.02	0.33±0.02	0.248	ns
Spine volume (a.u.)	0.20±0.01	0.18±0.01	0.057	ns
Spine length (µm)	1.56±0.08	1.95±0.09	0.0002	***
Head length (µm)	1.21±0.07	1.46±0.08	0.128	ns
Head width (µm)	0.48±0.03	0.45±0.03	0.642	ns

Spine volume and density: N≥200 spines per neuron, 5 neurons per group and experiment, 3 independent experiments. Spine morphology: N≥15 spines per neuron, 5 neurons per group and experiment, 3 independent experiments. ns: P≥0.05, ***: P<0.001.

Table S6E. Data shown in Fig. 6B-F

	CTR + CTR-sh	CTR + INF2-sh	P	

Spine density (μm^{-1})	0.50±0.02	0.34±0.02	<0.0001	****
Spine volume (a.u.)	0.23±0.01	0.24±0.01	0.11	ns
Spine length (μm)	1.58±0.10	1.53±0.08	0.67	ns
Head length (μm)	1.00±0.10	0.92±0.07	0.52	ns
Head width (μm)	0.51±0.02	0.49±0.02	0.49	ns

Spine volume and density: N≥200 spines per neuron, 5 neurons per group and experiment, 3 independent experiments. Spine morphology: N≥15 spines per neuron, 5 neurons per group and experiment, 3 independent experiments. ns: P≥0.05, ****: P<0.0001.

Table S6F. Data shown in Fig. 6I-M

	CTR + GFP	CTR + INF2-GFP	P	
Spine density (μm^{-1})	0.41±0.02	0.32±0.01	0.0001	****
Spine volume (a.u.)	0.21±0.01	0.16±0.01	<0.0001	***
Spine length (μm)	1.30±0.05	1.63±0.10	0.009	**
Head length (μm)	0.77±0.06	1.21±0.10	0.002	**
Head width (μm)	0.61±0.03	0.54±0.03	0.07	ns

Spine volume and density: N≥200 spines per neuron, 5 neurons per group and experiment, 3 independent experiments. Spine morphology: N≥15 spines per neuron, 5 neurons per group and experiment, 3 independent experiments. ns: P≥0.05, **: P<0.01, ***: P<0.001, ****: P<0.0001.

Table S6G. Data shown in Fig. 7B-F

	dKO + GFP	dKO + INF2-GFP	P	
Spine density (μm^{-1})	0.32±0.02	0.31±0.02	0.98	ns
Spine volume (a.u.)	0.17±0.01	0.18±0.01	0.25	ns
Spine length (μm)	1.70±0.08	1.61±0.07	0.46	ns
Head length (μm)	1.33±0.07	1.33±0.09	0.98	ns
Head width (μm)	0.48±0.02	0.46±0.02	0.46	ns

Spine volume and density: N≥200 spines per neuron, 5 neurons per group and experiment, 3 independent experiments. Spine morphology: N≥15 spines per neuron, 5 neurons per group and experiment, 3 independent experiments. ns: P≥0.05.

Table S6H. Data shown in Fig. 7I-M

	dKO + CTR-sh	dKO + INF2-sh	P	
Spine density (μm^{-1})	0.30±0.01	0.40±0.01	<0.0001	****
Spine volume (a.u.)	0.19±0.01	0.21±0.01	0.12	ns
Spine length (μm)	1.52±0.08	1.46±0.07	0.61	ns
Head length (μm)	1.20±0.07	1.01±0.06	0.06	ns
Head width (μm)	0.49±0.03	0.63±0.03	0.001	**

Spine volume and density: N≥200 spines per neuron, 5 neurons per group and experiment, 3 independent experiments. Spine morphology: N≥15 spines per neuron, 5 neurons per group and experiment, 3 independent experiments. ns: P≥0.05, **: P<0.01, ***: P<0.001, ****: P<0.0001.

Table 6I. Data shown in Fig. S2B-C

	Cre-mut + CTR-sh	Cre + CAP2-sh	P	
Spine density (μm^{-1})	0.44±0.02	0.30±0.02	<0.0001	****
Spine volume (a.u.)	0.25±0.01	0.20±0.01	0.0003	***

N≥200 spines per neuron, 4 neurons per group and experiment, 3 independent experiments. ***: P<0.001, ****: P<0.0001.

Table 6J. Data shown in Fig. S3B-E

			MV±SEM	T-test	P	
Filopodia	Length (µm)	CTR<br>CAP1-KO<br>CAP2-KO<br>dKO	2.75±0.114<br>2.81±0.160<br>2.64±0.280<br>3.23±0.256	CTR vs CAP1-KO<br>CTR vs CAP2-KO<br>CTR vs dKO	0.917<br>0.467<br>0.115	ns<br>ns<br>ns
	Width (µm)	CTR<br>CAP1-KO<br>CAP2-KO<br>dKO	0.249±0.013<br>0.258±0.023<br>0.241±0.022<br>0.261±0.017	CTR vs CAP1-KO<br>CTR vs CAP2-KO<br>CTR vs dKO	0.583<br>0.358<br>0.209	ns<br>ns<br>ns
Thin	Length (µm)	CTR<br>CAP1-KO<br>CAP2-KO<br>dKO	1.241±0.068<br>1.360±0.079<br>1.262±0.060<br>1.261±0.088	CTR vs CAP1-KO<br>CTR vs CAP2-KO<br>CTR vs dKO	0.278<br>0.828<br>0.862	ns<br>ns<br>ns
	Head length (µm)	CTR<br>CAP1-KO<br>CAP2-KO<br>dKO	0.810±0.061<br>0.774±0.064<br>0.698±0.041<br>0.729±0.079	CTR vs CAP1-KO<br>CTR vs CAP2-KO<br>CTR vs dKO	0.697<br>0.156<br>0.437	ns<br>ns<br>ns
	Head width (µm)	CTR<br>CAP1-KO<br>CAP2-KO<br>dKO	0.290±0.013<br>0.333±0.024<br>0.341±0.021<br>0.277±0.017	CTR vs CAP1-KO<br>CTR vs CAP2-KO<br>CTR vs dKO	0.141<br>0.056<br>0.558	ns<br>ns<br>ns
Stubby	Length (µm)	CTR<br>CAP1-KO<br>CAP2-KO<br>dKO	0.531±0.035<br>0.714±0.060<br>0.567±0.031<br>0.457±0.038	CTR vs CAP1-KO<br>CTR vs CAP2-KO<br>CTR vs dKO	0.017<br>0.470<br>0.185	*<br>ns<br>ns
	Width (µm)	CTR<br>CAP1-KO<br>CAP2-KO<br>dKO	0.588±0.035<br>0.788±0.072<br>0.654±0.042<br>0.526±0.057	CTR vs CAP1-KO<br>CTR vs CAP2-KO<br>CTR vs dKO	0.022<br>0.248<br>0.361	*<br>ns<br>ns
Mushroo m	Length (µm)	CTR<br>CAP1-KO<br>CAP2-KO<br>dKO	1.383±0.088<br>1.339±0.187<br>1.480±0.074<br>1.262±0.096	CTR vs CAP1-KO<br>CTR vs CAP2-KO<br>CTR vs dKO	0.646<br>0.427<br>0.376	ns<br>ns<br>ns

	Head length (µm)	CTR<br>CAP1-KO<br>CAP2-KO<br>dKO	0.723±0.047<br>0.600±0.081<br>0.640±0.042<br>0.540±0.030	CTR vs CAP1-KO<br>CTR vs CAP2-KO<br>CTR vs dKO	0.180<br>0.211<br>0.003	ns<br>ns<br>*
	Head width (µm)	CTR<br>CAP1-KO<br>CAP2-KO<br>dKO	0.799±0.032<br>0.876±0.081<br>0.857±0.033<br>0.861±0.058	CTR vs CAP1-KO<br>CTR vs CAP2-KO<br>CTR vs dKO	0.550<br>0.261<br>0.376	ns<br>ns<br>ns

N≥15 spines per neuron, 5 neurons per group and experiment, 3 independent experiments. ns: P≥0.05, *: P<0.05.

Table 6K. Data shown in Fig. S4A-D

		CTR (DIV11)	dKO (DIV11)	P	
**Filopodia**	Length (µm)	3.32±0.11	3.61±0.05	0.053	ns
	Width (µm)	0.25±0.01	0.26±0.01	0.79	ns
**Thin**	Length (µm)	1.23±0.01	1.23±0.03	0.90	ns
	Head length (µm)	0.94±0.03	0.84±0.09	0.45	ns
	Head width (µm)	0.26±0.01	0.27±0.02	0.32	ns
**Stubby**	Length (µm)	0.50±0.01	0.44±0.01	0.17	ns
	Width (µm)	0.56±0.06	0.60±0.04	0.96	ns
**Mushroom**	Length (µm)	1.45±0.04	1.62±0.17	0.49	ns
	Head length (µm)	0.65±0.06	0.48±0.02	0.10	ns
	Head width (µm)	0.84±0.06	0.86±0.05	0.84	ns

N ≥ 15 spines per neuron, 5 neurons per group and experiment, 3 independent experiments. ns: P≥0.05.

Table 6L. Data shown in Fig. S6C-F

		CTR + CTR-sh	CTR + INF2-sh	P	
**Filopodia**	Length (µm)	3.30±0.13	2.68±0.15	0.11	ns
	Width (µm)	0.37±0.01	0.38±0.02	0.84	ns
**Thin**	Length (µm)	1.15±0.05	1.20±0.06	0.23	ns
	Head length (µm)	0.91±0.05	0.86±0.06	0.34	ns
	Head width (µm)	0.34±0.01	0.33±0.01	0.66	ns
**Stubby**	Length (µm)	0.46±0.02	0.44±0.03	0.51	ns
	Width (µm)	0.49±0.02	0.49±0.03	0.87	ns
**Mushroom**	Length (µm)	1.76±0.09	1.87±0.08	0.47	ns
	Head length (µm)	0.74±0.04	0.73±0.03	0.89	ns
	Head width (µm)	0.75±0.03	0.74±0.03	0.82	ns

N ≥ 15 spines per neuron, 5 neurons per group and experiment, 3 independent experiments. ns: P≥0.05.

Table 6M. Data shown in Fig. S6G-J

		CTR + GFP	CTR + INF2-GFP	P	
**Filopodia**	Length (µm)	2.85±0.14	3.05±0.21	0.26	ns
	Width (µm)	0.32±0.02	0.35±0.01	0.44	ns

**Thin**	Length (µm)	0.99±0.04	1.05±0.08	0.52	ns
	Head length (µm)	0.87±0.05	0.92±0.08	0.61	ns
	Head width (µm)	0.34±0.01	0.35±0.01	0.72	ns
**Stubby**	Length (µm)	0.37±0.02	0.40±0.02	0.29	ns
	Width (µm)	0.40±0.02	0.43±0.03	0.31	ns
**Mushroom**	Length (µm)	1.51±0.16	1.64±0.13	0.33	ns
	Head length (µm)	0.70±0.05	0.67±0.03	0.78	ns
	Head width (µm)	0.69±0.03	0.66±0.04	0.83	ns

N ≥ 15 spines per neuron, 5 neurons per group and experiment, 3 independent experiments. ns: P≥0.05.

Table 6N. Data shown in Fig. S7A-D

		**dKO + GFP**	**dKO + INF2-GFP**	**P**	
**Filopodia**	Length (µm)	3.32±0.12	3.43±0.20	0.57	ns
	Width (µm)	0.35±0.03	0.30±0.01	0.66	ns
**Thin**	Length (µm)	1.20±0.06	1.10±0.05	0.23	ns
	Head length (µm)	1.04±0.05	0.99±0.06	0.54	ns
	Head width (µm)	0.28±0.01	0.27±0.01	0.66	ns
**Stubby**	Length (µm)	0.38±0.02	0.41±0.02	0.31	ns
	Width (µm)	0.58±0.02	0.65±0.04	0.18	ns
**Mushroom**	Length (µm)	1.49±0.10	1.51±0.10	0.90	ns
	Head length (µm)	0.52±0.03	0.49±0.04	0.49	ns
	Head width (µm)	0.90±0.05	0.84±0.04	0.38	ns

N ≥ 15 spines per neuron, 5 neurons per group and experiment, 3 independent experiments. ns: P≥0.05.

Table 6O. Data shown in Fig. S7E-H

		**dKO + CTR-sh**	**dKO + INF2-sh**	**P**	
**Filopodia**	Length (µm)	3.34±0.09	3.28±0.17	0.43	ns
	Width (µm)	0.25±0.01	0.27±0.02	0.34	ns
**Thin**	Length (µm)	1.08±0.05	1.22±0.05	0.07	ns
	Head length (µm)	0.90±0.03	1.08±0.05	0.01	*
	Head width (µm)	0.28±0.01	0.30±0.02	0.48	ns
**Stubby**	Length (µm)	0.42±0.03	0.45±0.03	0.54	ns
	Width (µm)	0.61±0.05	0.79±0.06	0.048	*
**Mushroom**	Length (µm)	1.34±0.12	1.51±0.12	0.30	ns
	Head length (µm)	0.44±0.03	0.59±0.05	0.02	*
	Head width (µm)	0.87±0.04	0.89±0.04	0.73	ns

N ≥ 15 spines per neuron, 5 neurons per group and experiment, 3 independent experiments. ns: P≥0.05, *: P<0.05.
