# Supplementary material for: Cyclase-associated protein (CAP) inhibits inverted formin 2 (INF2) to induce dendritic spine maturation
Source: Cell Mol Life Sci. 2024 Aug 18;81(1):353. doi: 10.1007/s00018-024-05393-y (PMC11335277; doi:10.1007/s00018-024-05393-y)
Supplement: Supplementary file 7 — Supplementary file7 Table S7. Sequences of CTR-sh and shRNA against either CAP2 or INF2 (PDF 68 KB) [file 18_2024_5393_MOESM7_ESM.pdf]

Table S7. Sequences of shRNA

Name	**Sequence (antisense)**
CTR-sh	CCUAAGGACCACAAGGUUU
CAP2-sh2	UUAUCCUCAUUCUCAAAGG
CAP2-sh3	UAUUCCACUCUCCACUUCU
CAP2-sh4	UAUGUCUUCUUGUCAUCUG
CAP2-sh1	GUAGGUGGGUGCUCUGUCC
CAP2-sh2	CUUGAGGGACGUUUCUUCC
CAP2-sh3	UAGGAUUGCUGGCAGUUGC
CAP2-sh4	UAUUGCUAGAGAACUCGAG
