# Supplementary figures and images for: Cyclase-associated protein (CAP) inhibits inverted formin 2 (INF2) to induce dendritic spine maturation
Source: Cell Mol Life Sci. 2024 Aug 18;81(1):353. doi: 10.1007/s00018-024-05393-y (PMC11335277; doi:10.1007/s00018-024-05393-y)

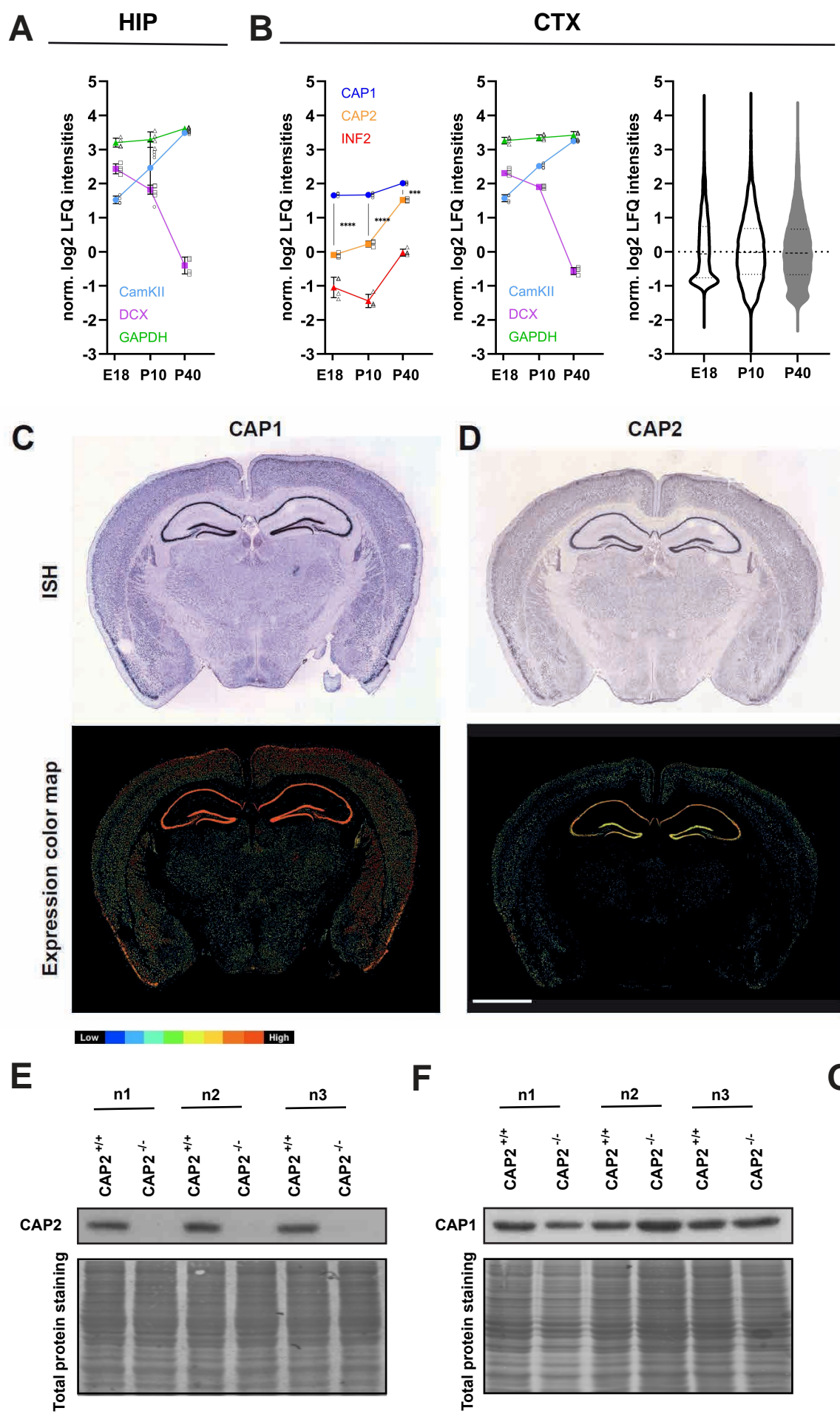

**Figure S1**

Supplement: Supplementary file 8 — Supplementary file8 Figure S1. (A) This plot supplements mass spectrometry data shown in Fig. 1B and shows the expected increase of CamKII expression and the expected decrease of doublecortin (DCX) expression throughout hippocampal development. Further, it includes expression levels of the house keeping protein glyceraldehyde 3-phosphate dehydrogenase (GAPDH). (B) Expression levels of CAP1, CAP2, INF2, CamKII, DCX and GAPDH determined by mass spectrometry on cerebral cortex lysates from E18.5, P10 and P40 mice. Violin plots to the right show expression level distribution of all proteins in these lysates. Norm. log2 LFQ intensity of zero indicates median expression level of all proteins detected within respective condition. Graph includes values of individual protein samples (N=4), MV±SEM. Statistical comparison of CAP1 and CAP2 expression levels were performed using Student’s t-test and corrected for multiple comparison with Bonferroni method. In situ hybridization and expression color map showing expression of (C) CAP1 and (D) CAP2 in the hippocampus and cerebral cortex from adult mice. Data were extracted from the Allen Mouse Brain Atlas, URLs of the images are provided in Table S3. Immunoblots with antibodies against (E) CAP2 and (F) CAP1 in hippocampal lysates of three adult CAP2-/- mice and CAP2+/+ littermates each. Total protein staining was performed to confirm equal loading. (G) Quantification of CAP1 levels in lysates from CAP2+/+ and CAP2-/- mice normalized to total protein load. Scale bar (mm): 3. ns: P>0.05, ***: P<0.001, ****: P<0.0001 (PDF 2976 KB) [file 18_2024_5393_MOESM8_ESM.pdf]

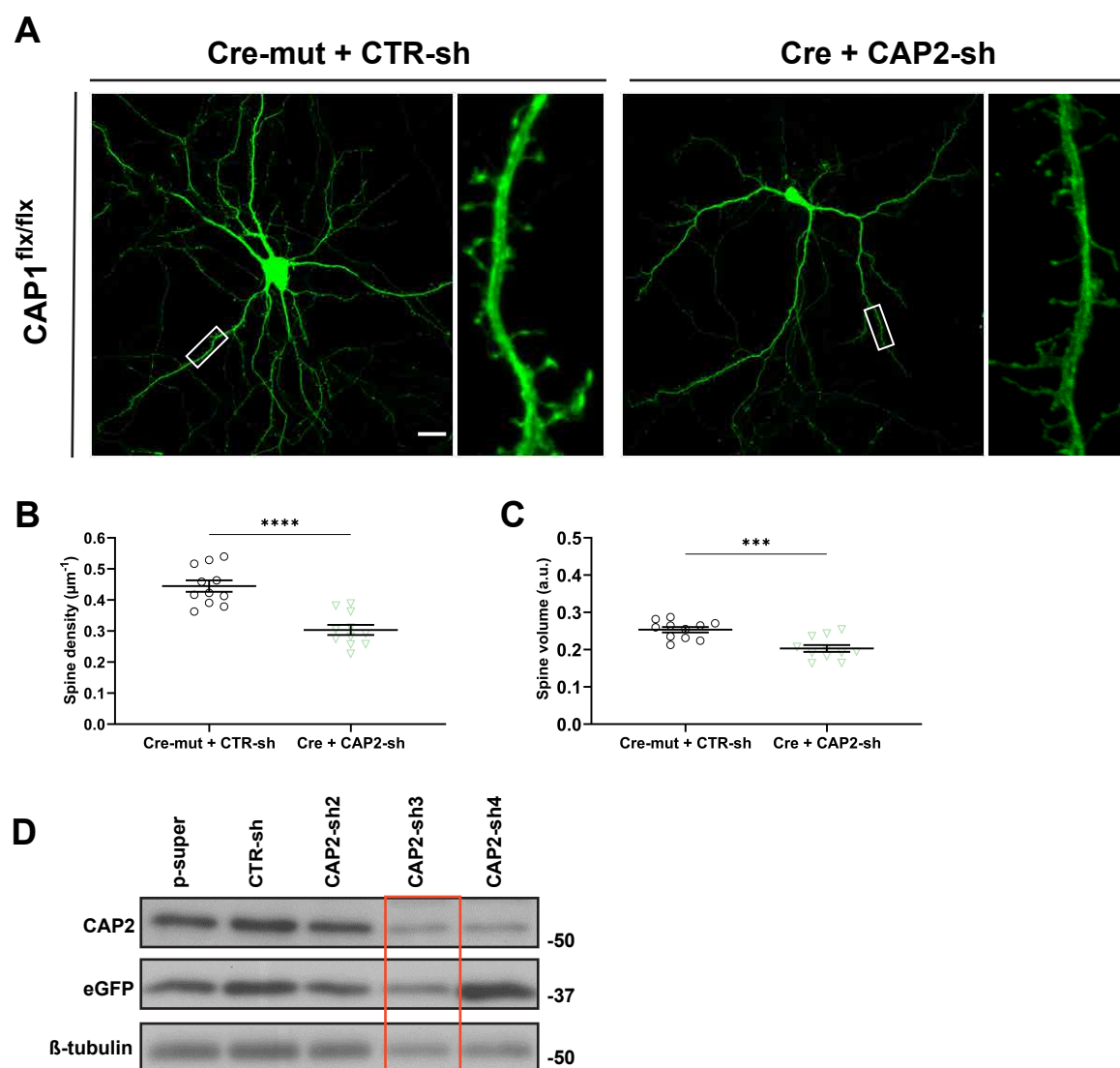

**Figure S2**

Supplement: Supplementary file 9 — Supplementary file9 Figure S2. (A) Micrographs of GFP-expressing CAP1flx/flx neurons transfected with either Cre-mut and CTR-sh or Cre and CAP2-sh3. Boxes indicate areas shown at higher magnification. Graphs showing (B) spine density and (C) spine volume in neurons transfected with either Cre-mut and CTR-sh or Cre and CAP2-sh. (D) Immunoblots showing CAP2 expression in lysates of cortical neurons upon electroporation of Ctr-sh and three different shRNAs against CAP2. Neurons were co-electroporated with GFP. GFP and β-tubulin were used as loading controls. Red box highlights CAP2-sh3, that has been used for CAP2 knockdown in morphometric analyses (Fig. S2A-C). Scale bar (µm): 20. ***: P<0.001, ****: P<0.0001 (PDF 609 KB) [file 18_2024_5393_MOESM9_ESM.pdf]

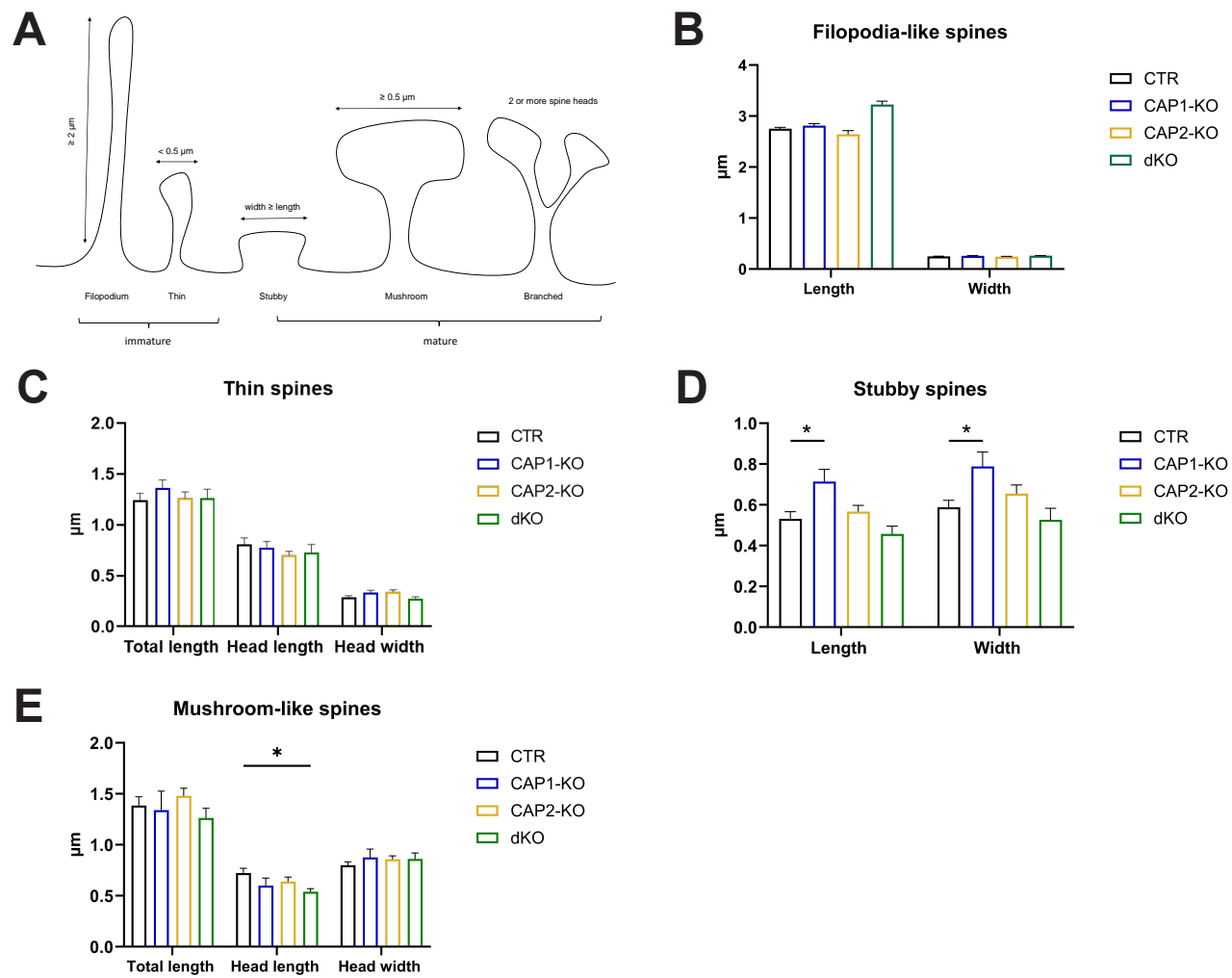

**Figure S3**

Supplement: Supplementary file 10 — Supplementary file10 Figure S3. (A) Scheme showing categorization of spine types. Graphs showing (B) length and width of filopodia-like spines, (C) length, head length and head width of thin spines, (D) length and width of stubby spines as well as (E) length, head length and head width of mushroom-like spines in CTR, CAP1-KO, CAP2-KO and dKO neurons. ns: P≥0.05, *: P<0.05 (PDF 1168 KB) [file 18_2024_5393_MOESM10_ESM.pdf]

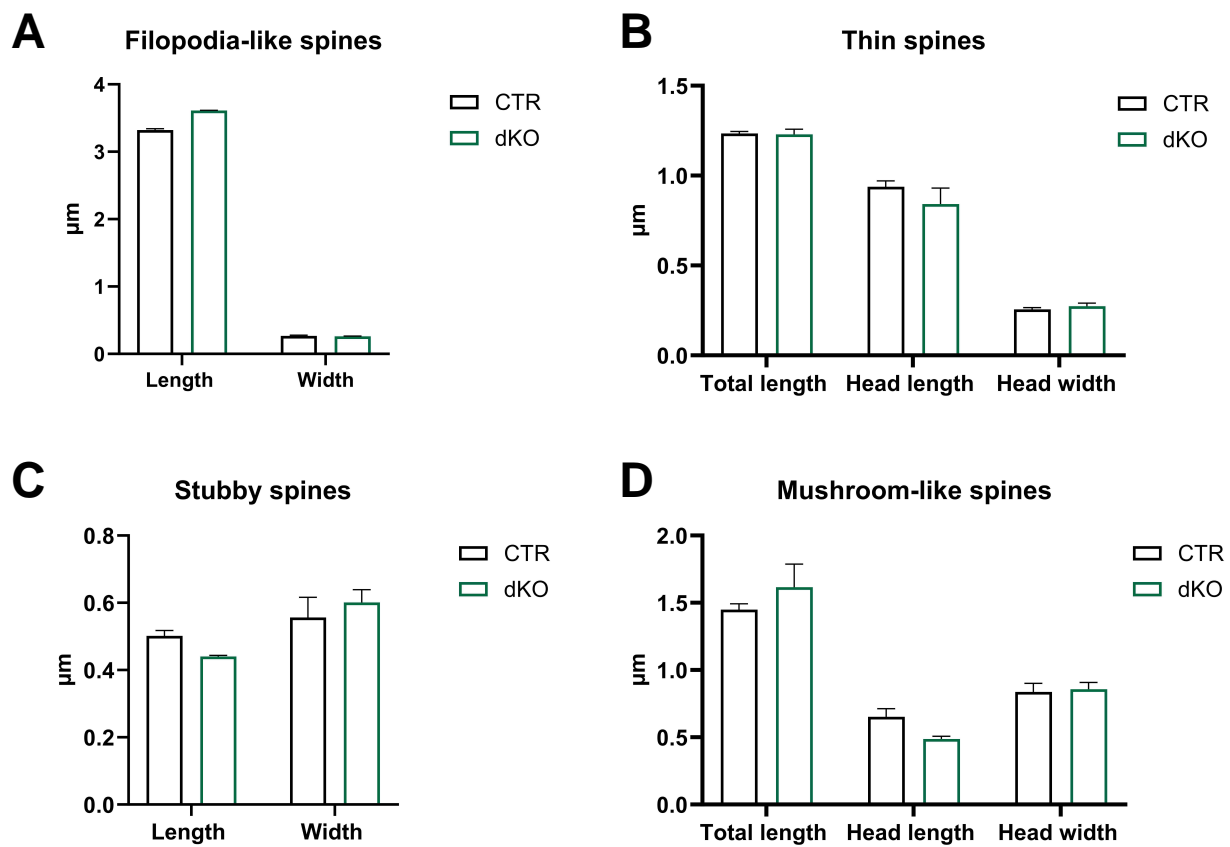

**Figure S4**

Supplement: Supplementary file 11 — Supplementary file11 Figure S4. Graphs showing (A) length and width of filopodia-like spines, (B) length, head length and head width of thin spines, (C) length and width of stubby spines as well as (D) length, head length and head width of mushroom-like spines in DIV11 CTR and dKO neurons (PDF 1062 KB) [file 18_2024_5393_MOESM11_ESM.pdf]

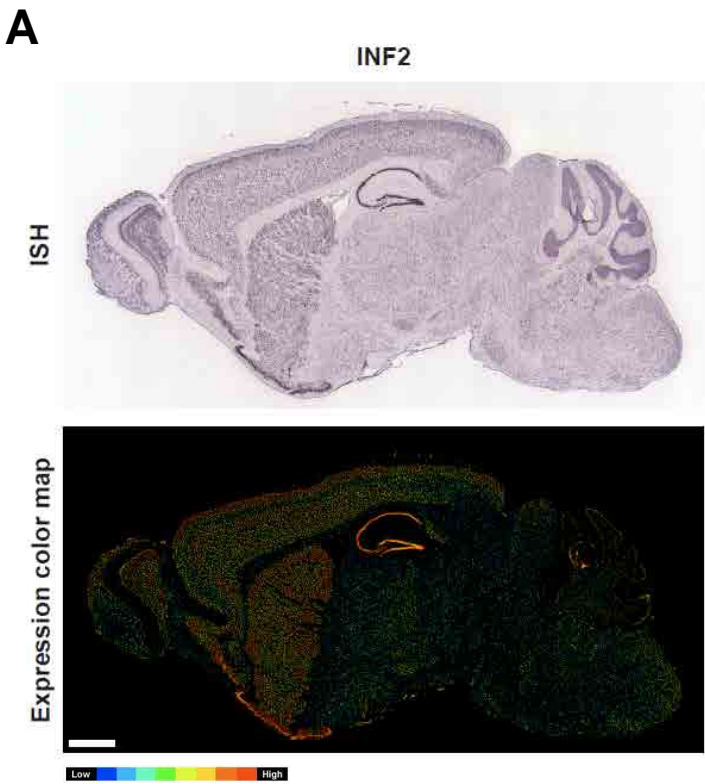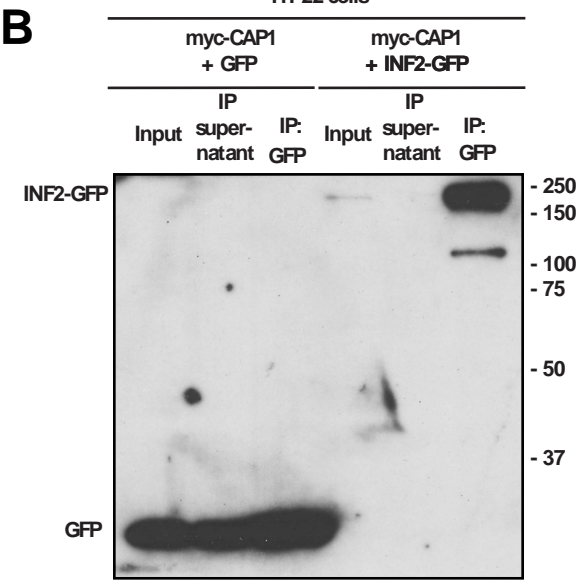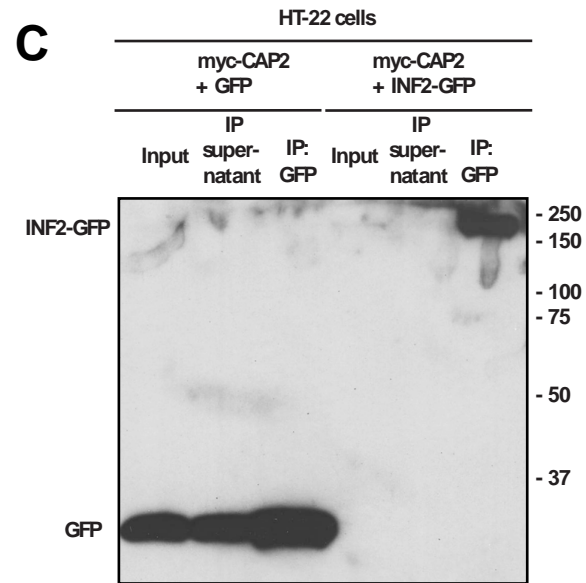

Figure S5

Supplement: Supplementary file 12 — Supplementary file12 Figure S5. (A) In situ hybridization and expression color map showing INF2 expression in the hippocampus, cerebral cortex and striatum from adult mice. Data were extracted from the Allen Mouse Brain Atlas, URL of the image is provided in Table S3. Immunoblots with antibody against GFP in lysates from HT-22 cells expressing (B) myc-CAP1 together with either GFP or INF2-GFP or (C) myc-CAP2 together with either GFP or INF2-GFP. The expression levels of INF2-GFP protein was rather low, but still detectable as a weak band in the input lane. Total protein staining as loading control for both immunoblots is shown in Fig. 5F-G. Scale bar (mm): 1.4 (PDF 1562 KB) [file 18_2024_5393_MOESM12_ESM.pdf]

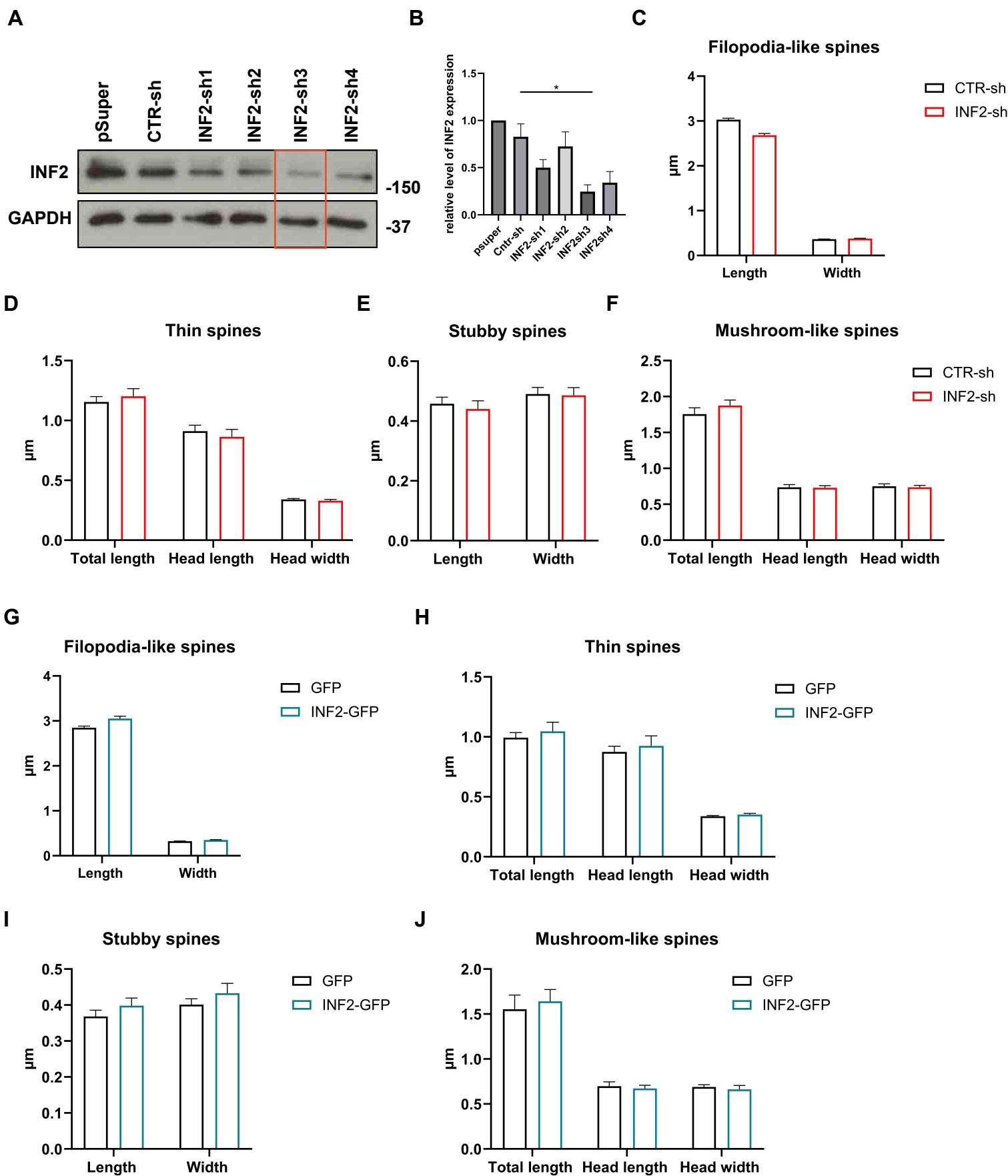

**Figure S6**

Supplement: Supplementary file 13 — Supplementary file13 Figure S6. (A) Immunoblot showing INF2 expression in HT-22 cells upon expression of CTR-sh or four different shRNA against INF2. GAPDH was used as loading control. Red box highlights INF2-sh3, that has been used for INF2 knockdown in morphometric analyses (Figs. 6A-G, 7H-N). (B) Quantification of INF2 in HT-22 cells expressing CTR-sh or different INF2-sh. N=immunoblots of three independent experiments. Graphs showing (C) length and width of filopodia-like spines, (D) length, head length and head width of thin spines, (E) length and width of stubby spines as well as (F) length, head length and head width of mushroom-like spines in DIV16 CTR neurons expressing either CTR-sh or INF2-sh. (G) Graphs showing (G) length and width of filopodia-like spines, (H) length, head length and head width of thin spines, (I) length and width of stubby spines as well as (J) length, head length and head width of mushroom-like spines in DIV16 CTR neurons expressing either GFP or INF2-GFP. *: P<0.05 (PDF 2447 KB) [file 18_2024_5393_MOESM13_ESM.pdf]

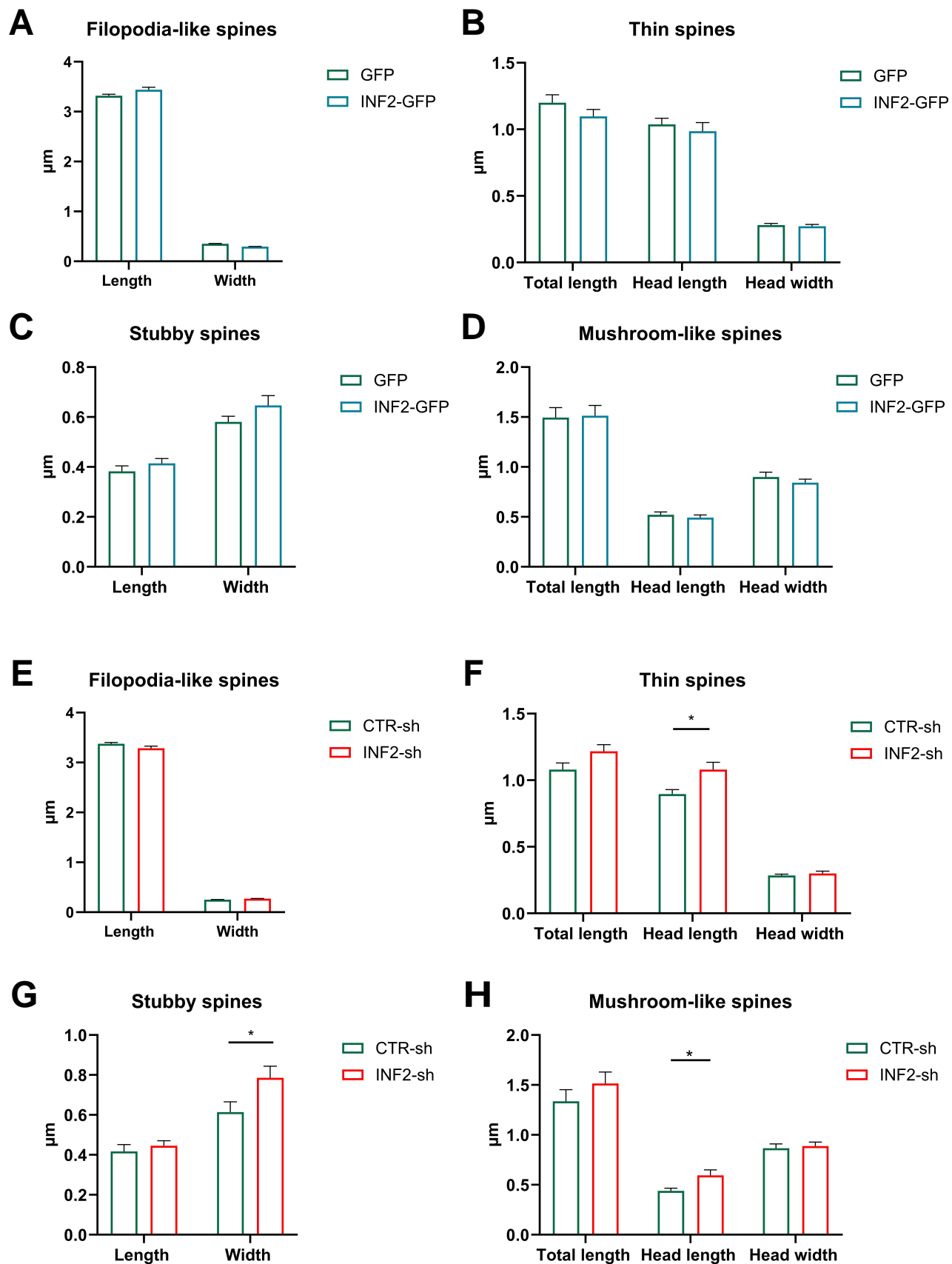

**Figure S7**

Supplement: Supplementary file 14 — Supplementary file14 Figure S7. Graphs showing (A) length and width of filopodia-like spines, (B) length, head length and head width of thin spines, (C) length and width of stubby spines as well as (D) length, head length and head width of mushroom-like spines in DIV16 dKO neurons expressing either GFP or INF2-GFP. (E) Graphs showing (G) length and width of filopodia-like spines, (H) length, head length and head width of thin spines, (I) length and width of stubby spines as well as (J) length, head length and head width of mushroom-like spines in DIV16 dKO neurons expressing either CTR-sh or INF2-sh (PDF 2305 KB) [file 18_2024_5393_MOESM14_ESM.pdf]
